# KDM5A and KDM5B histone-demethylases contribute to HU-induced replication stress response and tolerance

**DOI:** 10.1242/bio.057729

**Published:** 2021-05-26

**Authors:** Solenne Gaillard, Virginie Charasson, Cyril Ribeyre, Kader Salifou, Marie-Jeanne Pillaire, Jean-Sebastien Hoffmann, Angelos Constantinou, Didier Trouche, Marie Vandromme

**Affiliations:** 1 MCD, Centre de Biologie Integrative (CBI), University of Toulouse, CNRS, UPS, 31062 Toulouse, France; 2 Institut de Génétique Humaine, CNRS, Université de Montpellier, Montpellier, France; 3 Cancer Research Center of Toulouse, INSERM U1037, CNRS ERL5294, University of Toulouse 3, 31037 Toulouse, France; 4 Laboratoire de Pathologie, Institut Universitaire du Cancer-Toulouse, Oncopole, 1 avenue Irène-Joliot-Curie, 31059 Toulouse cedex, France

**Keywords:** Chromatin, Drug tolerance, Replication stress

## Abstract

KDM5A and KDM5B histone-demethylases are overexpressed in many cancers and have been involved in drug tolerance. Here, we describe that KDM5A, together with KDM5B, contribute to replication stress (RS) response and tolerance. First, they positively regulate RRM2, the regulatory subunit of ribonucleotide reductase. Second, they are required for optimal levels of activated Chk1, a major player of the intra-S phase checkpoint that protects cells from RS. We also found that KDM5A is enriched at ongoing replication forks and associates with both PCNA and Chk1. Because RRM2 is a major determinant of replication stress tolerance, we developed cells resistant to HU, and show that KDM5A/B proteins are required for both RRM2 overexpression and tolerance to HU. Altogether, our results indicate that KDM5A/B are major players of RS management. They also show that drugs targeting the enzymatic activity of KDM5 proteins may not affect all cancer-related consequences of KDM5A/B overexpression.

## Abstract (precis)

**Summary:** We describe that KDM5A and KDM5B histone-demethylases contribute to hydroxyurea-induced replication stress tolerance, in part by regulating expression of ribonuclease reductase subunit RRM2.

## INTRODUCTION

KDM5 proteins belong to the JUMONJI family of demethylases that catalyse the removal of methyl groups from lysine residues on histone tails. In mammals, the KDM5 subfamily includes four members, KDM5A (JARID1A/RBP2), KDM5B (JARID1B/PLU-1), KDM5C (JARID1C/SMCX) and KDM5D (JARID1B/SMCY) (Christensen et al., 2007; Iwase et al., 2007; Klose et al., 2007; Yamane et al., 2007).

KDM5 proteins are highly specific towards di- and tri-methylated states of lysine 4 of histone H3 (H3K4me2 and me3), which are active marks of transcription found at promoters of actively transcribed genes, H3K4me1 being enriched at active enhancers. Accordingly, KDM5 family proteins have been mainly described as transcriptional repressors. They participate in multi-subunits co-repressor complexes containing also histone deacetylase (HDAC) activity (Li et al., 2011; Nishibuchi et al., 2014; van Oevelen et al., 2008; Pasini et al., 2008; Tahiliani et al., 2007). However, they can also function in some instances as co-activators. This positive effect on transcription may involve or not their enzymatic activity and can result from the ability of KDM5A or KDM5B to prevent spreading of H3K4 methylation into gene bodies (Hayakawa et al., 2007; Xie et al., 2011), or from binding of KDM5B or KDM5C at enhancer regions to maintain H3K4 mono-methylation levels (Kidder et al., 2014; Outchkourov et al., 2013; Shen et al., 2016). Rather surprisingly, KDM5A and KDM5B bind to promoters of actively transcribed genes enriched in H3K4me3 (Beshiri et al., 2010; Liu and Secombe, 2015; Lopez-Bigas et al., 2008), meaning that their demethylase activity is tightly regulated, and that they function to recruit other regulators to chromatin (DiTacchio et al., 2011; Liu and Secombe, 2015; Nishibuchi et al., 2014; Secombe et al., 2007).

Several reports describe a role for these enzymes in safeguarding genomic stability. KDM5A is involved in the repair of DNA double strand breaks by homologous recombination (Gong et al., 2017). KDM5B allows the recruitment of key repair factors by demethylating H3K4me3 at sites of DNA damage (Li et al., 2014). Interestingly, KDM5C inactivation triggers genomic instability in renal cancers by interfering with heterochromatin replication (Rondinelli et al., 2015a). KDM5C also regulates replication at euchromatic early origins (Rondinelli et al., 2015b). These two studies link the activity of KDM5 enzymes to the process of replication.

Because of their high proliferative rate and deregulated oncogenes, cancer cells exhibit chronic replication stress (RS) defined as any hindrance to progression of the replication fork. Replication stress triggers the intra-S checkpoint relying on the kinase ATR [ATM (*ataxia telangiectasia*-mutated)- and rad3-related] and its downstream effector kinase CHK1 (Checkpoint Kinase 1) that induces a S-phase arrest, allowing replication stress resolution and fork restart. This mechanism ensures that the DNA is faithfully duplicated, and only once, at each cell cycle (Gaillard et al., 2015). CHK1 is currently viewed as a pro-tumour gene since it is frequently overexpressed in tumours, helping cancer cells to fight against genomic instability, thus preventing cell death (Rundle et al., 2017; Zhang and Hunter, 2014).

Cancer cells have developed specific ways to cope with replication stress, such as the overexpression of RRM2. RRM2 is a subunit of ribonucleotide reductase (RNR), which catalyses the formation of deoxyribonucleotides from ribonucleotides, and is thus involved in the supply of dNTPs during S-phase (Aye et al., 2015). Induction of exogenous replication stress is an important therapeutic strategy against cancer cells. Hydroxyurea (HU), which induces a strong replication stress by inhibiting RRM2 function, is used as a therapeutic drug in some cancers (Hehlmann et al., 2003; Levin, 1992; Sterkers et al., 1998). Overexpression of RRM2 is linked to resistance to HU (Choy et al., 1988; McClarty et al., 1987).

KDM5A, KDM5B and KDM5C are implicated in oncogenesis (Blair et al., 2011; Harmeyer et al., 2017). KDM5A is overexpressed in several cancers including acute myeloid leukemia and lung cancers (Oser et al., 2019; Shokri et al., 2018), KDM5B in breast cancer and melanoma (Roesch et al., 2008; Yamane et al., 2007), and KDM5C in prostate cancers (Stein et al., 2014), as well as metastatic breast and gastric cancers (Xu et al., 2017). Noticeably, there is growing evidence for a causal role of KDM5 subfamily in human cancer cells chemoresistance. In particular, KDM5A and KDM5B are involved in the emergence of drug tolerant cells (Roesch et al., 2010; Sharma et al., 2010). CPI-455, a pan-KDM5 inhibitor decreases drug tolerance in various models of cancer (Hou et al., 2012; Vinogradova et al., 2016 ; Fang et al., 2016; Wang et al., 2015). Moreover, KDM5A and KDM5B participate in radioresistance since their depletion sensitizes cancer cells to DNA damages induced by radiation or radiomimetic compounds (Bayo et al., 2018; Lin et al., 2015).

Here we describe a role for KDM5A and KDM5B in managing the replication stress response by fine-tuning the expression of RRM2 in response to HU. Moreover, we report that KDM5A and KDM5B are required optimal CHK1 expression, and that KDM5A localizes at replication forks together with PCNA and CHK1, in unchallenged conditions. Importantly, we show that the acquired resistance of cells to HU depends on KDM5A- and to a lesser extent KDM5B-dependent upregulation of RRM2.

## RESULTS

### KDM5A and KDM5B positively regulate RRM2

**Fig. 1.**
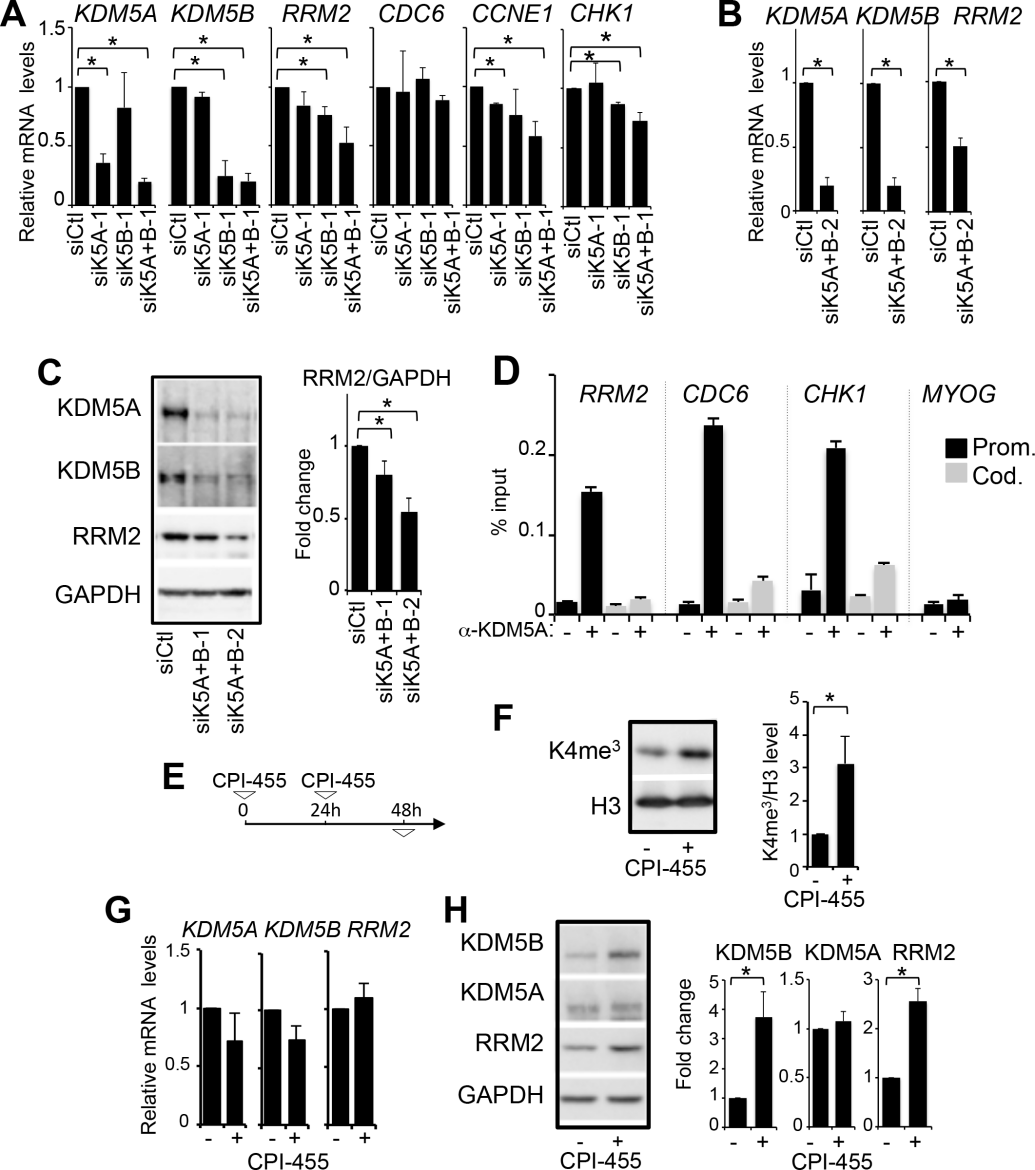
**KDM5A/B positively regulates RRM2 expression.** (A) Relative mRNA expression levels of KDM5A, KDM5B, RRM2, CDC6, CCNE1 and CHK1 upon transfection of the indicated siRNA in U2OS cells (siCtl corresponds to a non-targeting pool of siRNA). Expression levels were normalized to the reference gene P0 (ribosomal phosphoprotein P0) and calculated relative to 1 for the siCtl sample. mean±s.d., *n*=3 *P<0.05 (paired *t*-test) (B) Same as in A with another couple of KDM5A and KDM5B siRNAs. *P<0.05 (paired *t*-test). (C) Western blot analysis of KDM5A, KDM5B, RRM2 and GAPDH as a loading control from U2OS cells transfected with siRNA directed against KDM5A and KDM5B. Two distinct couples of siRNA (siK5A+B-1 and −2) were used. Quantification is shown in the right panel, following normalization to 1 for siCtl treated cells. mean±s.e.m., *n*=3. *P<0.05 (paired *t*-test). (D) ChIP analysis of KDM5A presence on the RRM2, CDC6 and CHK1 promoters (Prom.) and coding (Cod.) regions. The myogenin gene (MYOG) is not expressed in U2OS cells and its promoter serves as a negative control. A representative experiment out of three is shown. (E) Experimental design for CPI-455 treatments in panels F, G and H. (F) Western blot analysis of H3K4me3 and total H3 from U2OS cells treated with CPI-455 (+) or DMSO (−). A quantification of H3K4me3/H3 is shown in the right panel following normalization to 1 for siCtl cells. mean±s.e.m., *n*=3. *P<0.05 (paired *t*-test). (G) Relative mRNA expression levels of KDM5A, KDM5B and RRM2 in cells treated as in F. Expression levels were normalized to the reference gene P0 and calculated relative to 1 for the siCtl sample. mean±s.e.m., *n*=3. A paired *t*-test indicated no significant difference between CPI treated and untreated cells for the three genes. (H) Western blot analysis of KDM5A, KDM5B, RRM2 and GAPDH from U2OS cells treated as in F. Quantification is shown in the right panel following normalization to 1 for DMSO treated cells. mean±s.e.m., *n*=3. *P<0.05 (paired *t*-test).

Because KDM5A or/and KDM5B are involved in the stable repression of E2F-dependent genes during differentiation and senescence (Chicas et al., 2012; van Oevelen et al., 2008), we asked if they regulate these genes in proliferative U2OS cells. KDM5A or/and KDM5B were depleted using specific siRNA (siK5A, siK5B), and mRNA expression levels of the E2F targets CDC6, CCNE1, CHK1 and RRM2 were quantified 48 h later. Depletion of KDM5A or KDM5B alone had no or only minor effects on the expression of these genes (Fig. 1A). However, surprisingly given the known repressive role of KDM5A on E2F-regulated promoters, RRM2, CHK1, and CCNE1 expression were diminished when KDM5A and KDM5B were depleted together. In contrast, CDC6 was not affected, indicating that this effect is not observed on all E2F-target genes. Depletion of KDM5A and KDM5B using another couple of siRNA, also downregulated RRM2 expression ruling out off-target effects (Fig. 1B). Importantly, RRM2 protein expression was also decreased using two distinct sets of siRNA targeting KDM5A and KDM5B (Fig. 1C). Altogether, these data indicate that KDM5A and KDM5B favour the expression of RRM2, and suggest that they may compensate each other. Accordingly, by ChIP, KDM5A was found recruited to the RRM2 and CHK1 promoters, but not to their gene body nor to the promoter of a gene inactive in non-muscle cells (MYOG) (Fig. 1D). KDM5A was also recruited to the CDC6 promoter which is not affected by KDM5A and KDM5B depletion, consistent with the observation that KDM5A binds to promoters of actively transcribed genes but regulates only a subset of genes (Beshiri et al., 2012; Brier et al., 2017).

Next, we questioned if the demethylase activity of KDM5A and KDM5B is required for regulating RRM2 expression. We treated U2OS cells with CPI-455 (Fig. 1E), a specific inhibitor of KDM5 enzymatic activity (Vinogradova et al., 2016). As expected, this treatment increased H3K4me3 levels (Fig. 1F). KDM5A, KDM5B and RRM2 mRNA levels were not changed or weakly affected by CPI-455 treatment (Fig. 1G). KDM5A protein levels remained unchanged whereas both KDM5B and RRM2 protein levels were increased, probably by post-transcriptional events (Fig. 1H). Nevertheless, these data indicate that inhibiting KDM5A and KDM5B enzymatic activity does not recapitulate the effect of their depletion on RRM2 expression. Note, however, that given that CPI-455 treatment also inhibits KDM5C and KDM5D, we cannot formally rule out the possibility that the inhibition of KDM5C (KDM5D is not expressed in U2OS cells) compensates for KDM5A and KDM5B inhibition with respect to RRM2 expression.

Altogether, these data indicate that KDM5A and KDM5B favour the expression of RRM2, probably through binding to its promoter and in a demethylase-independent manner.

### KDM5A and KDM5B downregulation triggers endogenous replication stress in U2OS

**Fig. 2.**
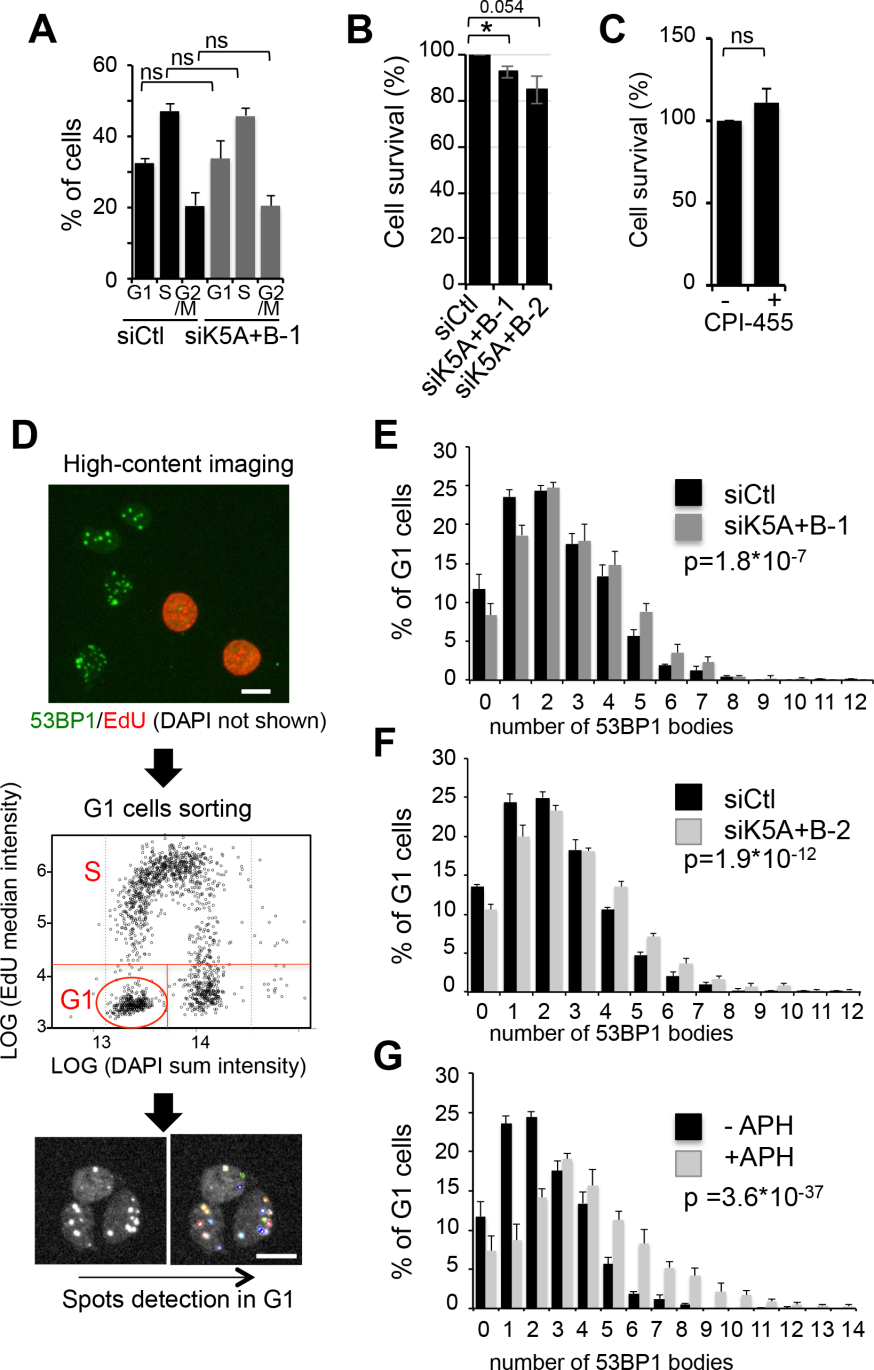
**KDM5A and KDM5B inhibition induces replicative stress.** (A) Cell cycle distribution of U2OS cells depleted for KDM5A and KDM5B using siK5A+B-1 compared to siCtl treated cells, analysed by the high content imaging system Operetta following EdU labelling and DAPI staining. mean±s.d., *n*=3. ns, non-significant (paired *t*-test). (B) Percentage of living cells following depletion of KDM5A and KDM5B using siK5A+B-1 or siK5A+B-2 siRNAs, following normalization to 100 for siCtl treated cells. mean±s.d., *n*=3. *P<0.05 or=0.054 (paired *t*-test). (C) Percentage of living cells following treatment each 24 h with CPI-455 (+) or DMSO (−) for 72 h and following normalization to 100 for DMSO treated cells. mean±s.d., *n*=3. ns, non-significant (paired *t*-test). (D) Schematic representation of the analysis used in E, F and G. (E) U2OS cells were transfected with siRNA directed against KDM5A and KDM5B (siK5A+B-1), or a non-targeting siRNA pool as control (siCtl) and stained for 53BP1, EdU (added in the medium 30 min before fixation) and DAPI. Images were acquired with the Operetta device and 53BP1 bodies were counted in G1 cells nuclei (sorted by EdU/DAPI staining), using the Colombus software. Number of G1 cells analysed was >700 for each condition. *P*-values of the difference to the control sample are indicated (Wilcoxon test). (F) Same as in E, except that another couple of KDM5A and KDM5B siRNAs was used. (G) Same as in E, except that cells were treated or not with 0.2 µM aphidicholin rather than transfected with siRNAs.

The genes we found affected by KDM5A and KDM5B depletion, CCNE1 and RRM2, encode important cell cycle regulators: CCNE1 is important for progression from G1 to S phase as the regulatory subunit of the CyclinE/cdk2 kinase complex. RRM2 is a subunit of RNR and is thus involved in the supply of dNTPs during S-phase. Despite the decrease of the expression of these two genes, the cell cycle distribution of U2OS cells was not disturbed upon depletion of KDM5A and KDM5B (Fig. 2A). Cell survival was slightly but significantly decreased (Fig. 2B), independently of histone demethylase activity, since no change in cell survival could be observed upon CPI-455 treatment (Fig. 2C).

Because RRM2 depletion leads to replication stress, we tested whether replication stress is induced upon KDM5A and KDM5B depletion, which would explain the decreased survival of cells. As a specific read-out for endogenous replication stress, we monitored 53BP1 bodies formation in G1. Indeed, replication stress results in the expression of common fragile sites which are regions of the genome hard to replicate. When incompletely replicated, these sites are found into 53BP1 bodies in the next G1 phase, waiting for the following S phase to be fully replicated (Harrigan et al., 2011; Lukas et al., 2011; Spies et al., 2019). 53BP1 bodies formation was monitored by high throughput microscopy as explained in Fig. 2D. The percentage of G1 cells with a high number of 53BP1 bodies increased upon depletion of KDM5A/B, whereas the number of cells with no or only one 53BP1 body decreased accordingly (Fig. 2E). Similar results were obtained using a distinct couple of siRNA (Fig. 2F). As a control, the number of G1 53BP1 foci increases with a low dose of aphidicolin, a condition known to increase 53BP1 bodies in G1 cells (Fig. 2G). Thus, depletion of KDM5A and B triggers endogenous replication stress, consistent with RRM2 downregulation.

### KDM5A and KDM5B restrain replication stress in response to HU

**Fig. 3.**
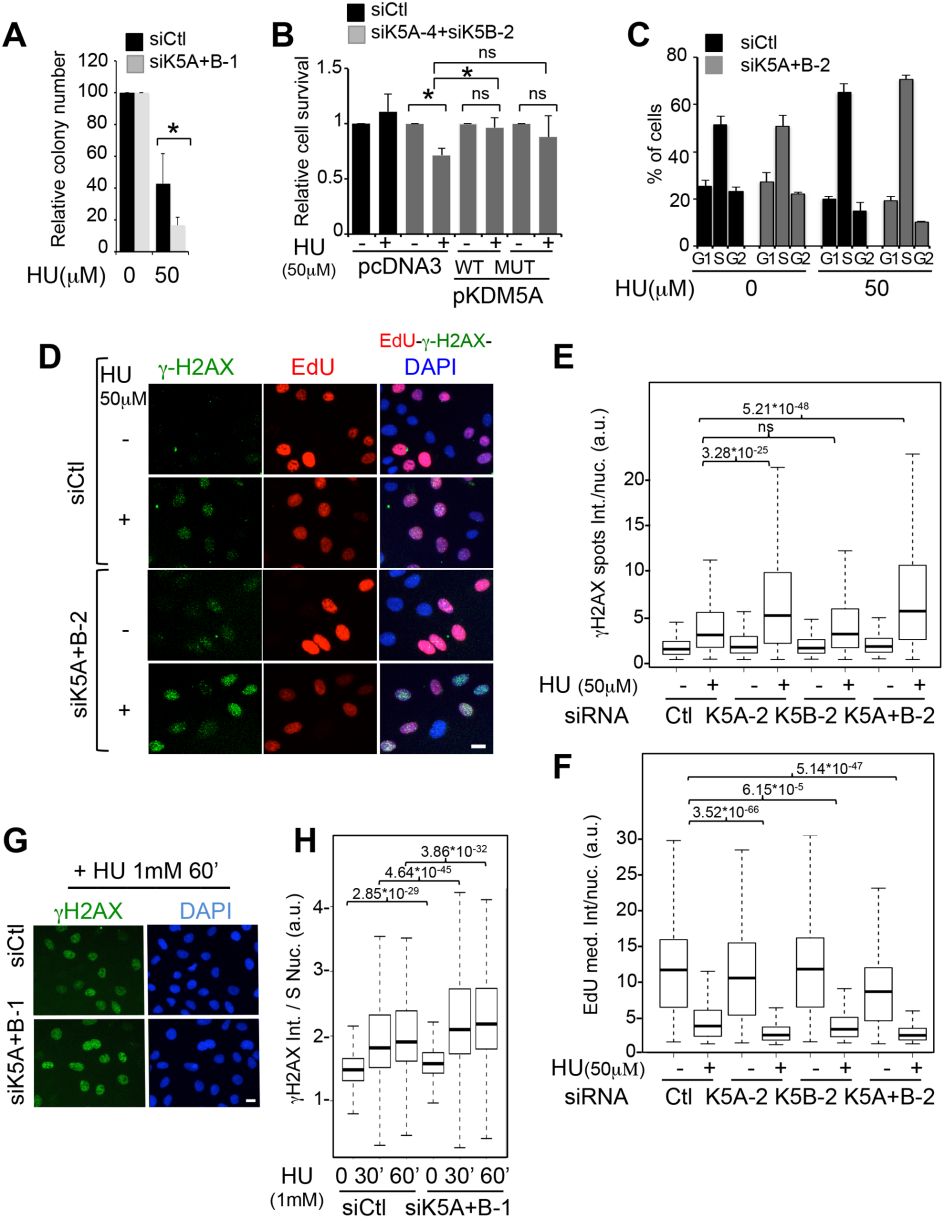
**KDM5A/B protect cells from HU-induced replication stress.** (A) Clonogenic assay of cells treated with siRNA directed against KDM5A-1 and KDM5B-1 or a non-targeting siRNA pool as control (siCtl), and exposed for 24 h to 50 µM HU, before allowing clones to grow for 10 days more. The number of clones was normalized to 100 for untreated siCtl cells. mean±s.e.m., *n*=3. *P<0.05 (paired *t*-test). (B) U2OS cells were electroporated with the indicated siRNA (siK5A-4 targets the 5′UTR of KDM5A) and 24 h later transfected with the indicated expression vector coding for wild-type KDM5A (pKDM5A WT) or a histone demethylase-defective mutant (MUT). To ensure efficient knockdown of KDM5A and KDM5B throughout the time course of the experiment, cells were transfected once more with siRNA 24 h following plasmids transfection, and cells were harvested and counted 24 h following this second siRNA transfection. The percentage of viable cells following normalization to 100 for untreated cells is represented. mean±s.e.m., *n*=4. *P<0.05 (paired *t*-test). ns, non-significant. (C) U2OS cells were electroporated with the indicated siRNA. 24 h later, they were incubated with 50 µM HU for further 24 h or left untreated. Cells were labelled with EdU during 30 min before fixation, stained for EdU and DAPI, and images were acquired using the operetta device. Cell cycle distribution was analysed thanks to the Colombus software. A representative experiment is shown. (D) Cells were transfected as in C, and treated with 50 µM HU for 24 h or left untreated. Cells were labelled with EdU during 30 min before fixation and stained for γ-H2AX, EdU incorporation, and DNA content by DAPI. Images were acquired using the high-content imaging device Operetta. Representative images from the experiment quantified in E and F are shown. Scale bar: 5 µM. (E) Cells were transfected by the indicated siRNA and treated or not with 50 µM HU, as indicated in C. γH2AX staining was quantified in the nuclei (spots total intensity) of S phase cells (sorted according to EdU/DAPI staining). A representative experiment out of three is shown. Results are presented as box-plots showing the median, the 25% and 75% quantiles and extrema. Number of S phase cells is >650 cells for each point. *P*-values are indicated (Wilcoxon). ns, non-significant. (F) Cells were transfected as in E, and EdU staining in S phase nuclei was measured. The median intensity of EdU per nucleus is represented as box-plots. Number of S phase cells >1000 for each point. *P*-values are indicated (Wilcoxon). (G) As in D, except that cells were treated with 1 mM HU for 0, 30, or 60 min as indicated. Cells were stained for γ-H2AX and DNA content by DAPI. Images were acquired using the operetta device. Representative images of cells from the 60 min point from the experiment quantified in H are shown. Scale bar: 5 µM. (H) Cells were treated as in G. Total nuclear intensity of γ-H2AX was quantified in nuclei of S phase U2OS cells, sorted according to DAPI staining, and presented as box-plots. A representative experiment out of three is shown. Number of S phase cells examined >1200 for each point. *P*-values are indicated (Wilcoxon).

Induction of replication stress is an important strategy of anti-cancer therapies. Given that KDM5A and KDM5B expression are often affected in human cancer, we next investigated whether KDM5A and KDM5B expression participate in the management of drug-induced replication stress. We induced replication stress using HU, which is used in anti-cancer therapy, and that functions as an inhibitor of RNR. By clonogenic assay, we found that treatment of cells with HU (50 µM) decreased the number of viable colonies, as expected. Interestingly, the co-depletion of KDM5A and KDM5B further diminished this number of clones (Fig. 3A). Moreover, KDM5A and KDM5B depletion decreased cell survival in response to sub-lethal doses of HU, and this decrease was reversed by the overexpression of wild-type KDM5A, concomitantly with an increase of RRM2 expression (Fig. 3B; see Fig. S1 for western blots monitoring KDM5A and RRM2 expression levels). Overexpression of enzymatic dead KDM5A did not induce a significant effect. Altogether, these data indicate that the expression of KDM5A and KDM5B favours cell survival in the presence of exogenous replication stress.

We next analysed the phosphorylation of H2AX (γ-H2AX), a hallmark of the DNA damage response (DDR). Using high content imaging, we quantified γH2AX spots intensity in S phase cells. EdU incorporation was also quantified to estimate the efficiency of ongoing replication. As expected, treatment with HU led to a block in S phase (Fig. 3C), and to an increase in nuclear γ-H2AX content in S phase cells’ nuclei (Fig. 3D,E). Depletion of KDM5A and B did not change cell cycle distribution (Fig. 3C). Strikingly, however, KDM5A and KDM5B depletion further increased the level of γ-H2AX induced by HU (Fig. 3D,E; see Fig. S2 for a statistical analysis from three independent experiments), accompanied by a decrease in EdU incorporation (Fig. 3F). Importantly, these results were reproduced with a distinct set of siRNA, excluding off-target effects (Fig. S2). These data suggest that KDM5A and KDM5B jointly protect cells from HU-induced replication stress, although we cannot exclude that they may have unshared specific activities. Indeed, although depletion of KDM5A and KDM5B together always leads to a more profound phenotype, KDM5A depletion induces significant increase in γ-H2AX staining and decrease in EdU incorporation (Fig. 3E and Fig. S2).

Because long term treatments with HU are known to induce fork collapse, and subsequent generation of DNA double strand breaks (Petermann et al., 2010; Saintigny et al., 2001), the results obtained above could reflect the described role of KDM5A and KDM5B in DNA breaks repair (Bayo et al., 2018; Gong et al., 2017). To investigate whether KDM5A and KDM5B play a direct role in the replication stress response, we looked for γ-H2AX signal intensity in nuclei of cells treated for shorter time (30 and 60 min) with 1 mM HU, conditions known to induce replication stress. At these concentrations of HU, replication is almost stopped and EdU cannot be used to follow cell cycle distribution. S phase cells were sorted out by DAPI stain intensity (Roukos et al., 2015). As shown in Fig. 3G and H, cells depleted for KDM5A and KDM5B displayed higher levels of γ-H2AX than control cells upon HU addition.

Altogether, these results suggest a role for KDM5A and KDM5B in protecting the genome from replication stress.

### KDM5A and KDM5B are required for full activation of Chk1 in response to RS

**Fig. 4.**
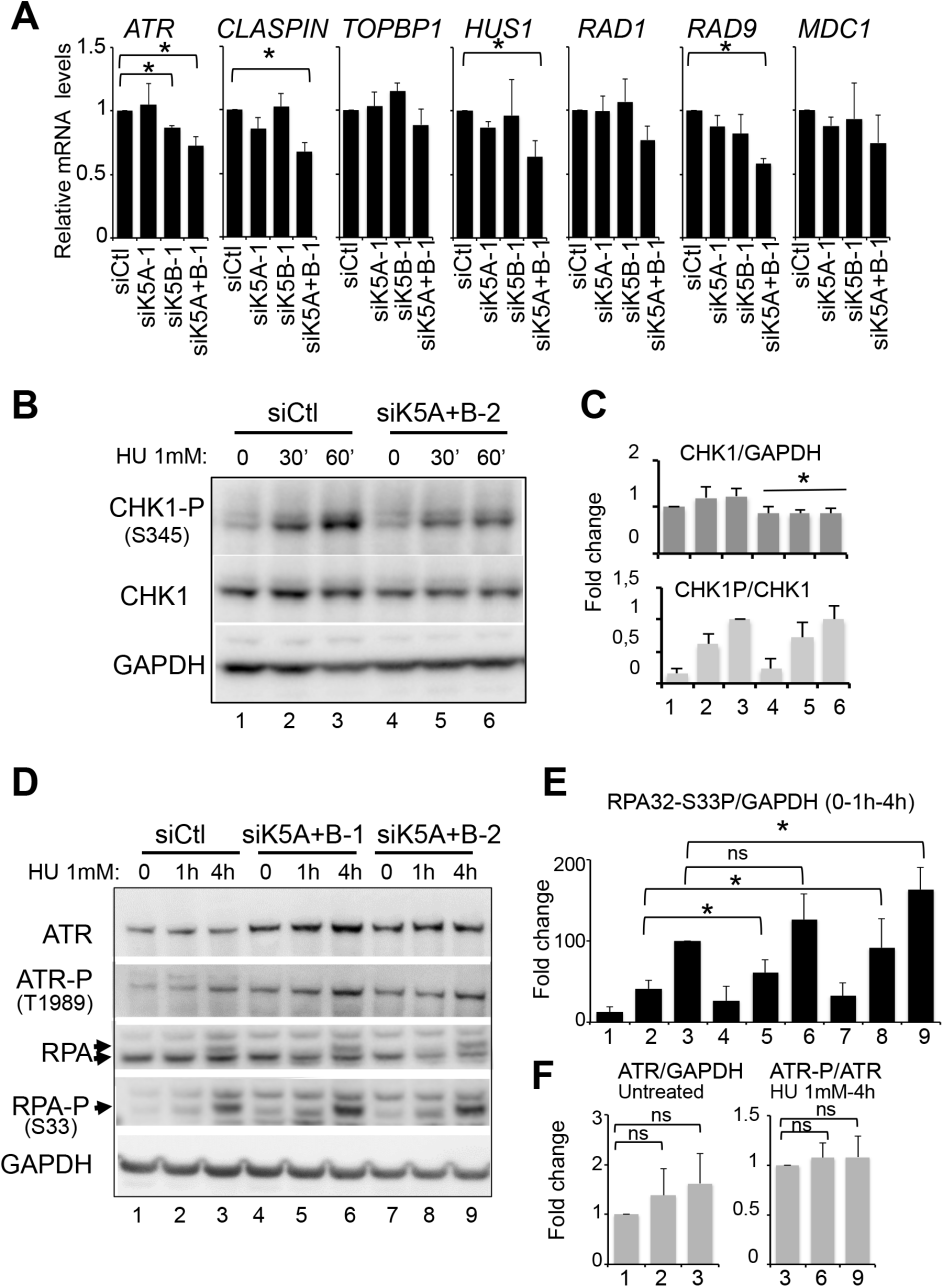
**KDM5A and B depletion affects the ATR-CHK1 pathway.** (A) Relative mRNA expression of several components of the ATR-CHK1 pathway (CHK1 is presented in Fig. 1A) in U2OS cells treated with siRNA directed against KDM5A or/and KDM5B or a non-targeting siRNA as control (siCtl). mRNA expression is normalized with the reference gene P0, and calculated relative to 1 for the siCtl. mean±s.d., *n*=3. *P<0.05 (paired *t*-test). (B) U2OS cells were transfected by the indicated siRNA, treated or not with 1 mM of HU for the indicated time and subjected to western blot analysis of KDM5A, KDM5B, CHK1 and S345-phospho CHK1 (CHK1-P) expression. GAPDH is used as a loading control. A representative western blot out of three is shown. (C) Quantifications of panel B for CHK1 normalized with GAPDH and CHK1-P normalized with CHK1, mean±s.e.m., *n*=3. *P<0.05 (paired *t*-test). Note that the same quantification is shown in Fig. S3 with individual siRNA transfection included. (D) U2OS cells were transfected by the indicated siRNA, treated or not with 1 mM of HU for the indicated time and subjected to western blot analysis of ATR, Ser1989-phospho ATR (ATR-P), S33-phospho RPA (RPA-P), and RPA expression levels. GAPDH serves as a loading control. A representative experiment is shown. (E,F) Quantifications of panel D for RPA-P relative to GAPDH (E), and for ATR relative to GAPDH and ATR-P relative to ATR (F), in untreated and after 4 h of treatment with 1 mM HU, respectively. mean±s.e.m., *n*=3. *P<0.05, ns, non-significant (paired *t*-test).

The response to RS mostly relies on the activation of the sensor kinase ATR, which phosphorylates itself on Thr1989 (Liu et al., 2011; Nam et al., 2011) and the effector kinase CHK1 on Ser345 (Liu et al., 2000), resulting in its activation. Results from Fig. 1A show that CHK1 expression is affected by KDM5A and KDM5B depletion, suggesting that KDM5A and KDM5B could regulate this pathway. To investigate further this possibility, we quantified mRNA expression levels of other key components of the ATR-CHK1 signalling, including TopBP1, the Rad9-Rad1-Hus1 (9-1-1) complex, and Claspin (Gaillard et al., 2015; Rundle et al., 2017; Zhang and Hunter, 2014). We found that *ATR*, *CLASPIN*, *HUS1*, *RAD9* mRNA were also decreased upon depletion of both KDM5A and KDM5B (Fig. 4A). Noticeably, depletion of KDM5B is sufficient to decrease *ATR* expression, but as observed for RRM2 and CHK1 (Fig. 1A), depletion of both KDM5A and KDM5B synergizes to affect expression of these genes.

We next analysed the effect of KDM5A and/or KDM5B depletion on activation of the ATR/CHK1 pathway. Upon treatment with 1 mM HU, CHK1 was rapidly phosphorylated on Ser345 (CHK1-P), reflecting its activation (Fig. 4B). Upon depletion of KDM5A and KDM5B together, CHK1 protein levels significantly decreased (Fig. 4B), as it was observed at mRNA levels (Fig. 1A). As a consequence, a diminution in the total amount of Ser345-phosphorylated CHK1 (CHK1-P) was also observed at the 60 min point after HU treatment. However, the ratio of CHK1-P to total CHK1 was similar (Fig. 4C) indicating that ATR signalling to CHK1 is not affected. Importantly, this was observed using another independent couple of siRNAs directed against KDM5A and KDM5B (Fig. S3). It has to be noted that depletion of either KDM5A or KDM5B did not have any significant effect on CHK1 expression and CHK1-P/CHK1 ratio (Fig. S3).

Although knocking-down KDM5A and KDM5B diminished ATR mRNA levels (Fig. 4A), ATR protein levels were not decreased [although a slight increase in protein could even be observed in Fig. 4D, quantification (*n*=3; Fig. 4F) indicated that this increase was not significant], suggesting a post-transcriptional control of ATR levels in these conditions. Moreover, KDM5A and KDM5B depletion did not impede phosphorylation of ATR on Thr1989, showing that ATR activation is not affected (Fig. 4D,F) (note that we had to treat cells for 4 h with HU to see a reproducible increase in ATR phosphorylation). RPA phosphorylation on Ser33 was slightly induced upon KDM5A and KDM5B depletion (Fig. 4D,E), as shown above for γH2AX. Thus, these data demonstrate that levels of activated CHK1 are diminished in KDM5A- and KDM5B-deficient cells, mainly because CHK1 expression is impaired. Note, however, that it is not sufficient to abolish the CHK1-dependent checkpoint since cells still accumulated in S phase when treated with HU. Altogether, our results thus indicate that KDM5A and KDM5B are needed for optimal CHK1 expression, but are not required for its activation by ATR.

### KDM5A localizes to the fork and interacts with PCNA

**Fig. 5.**
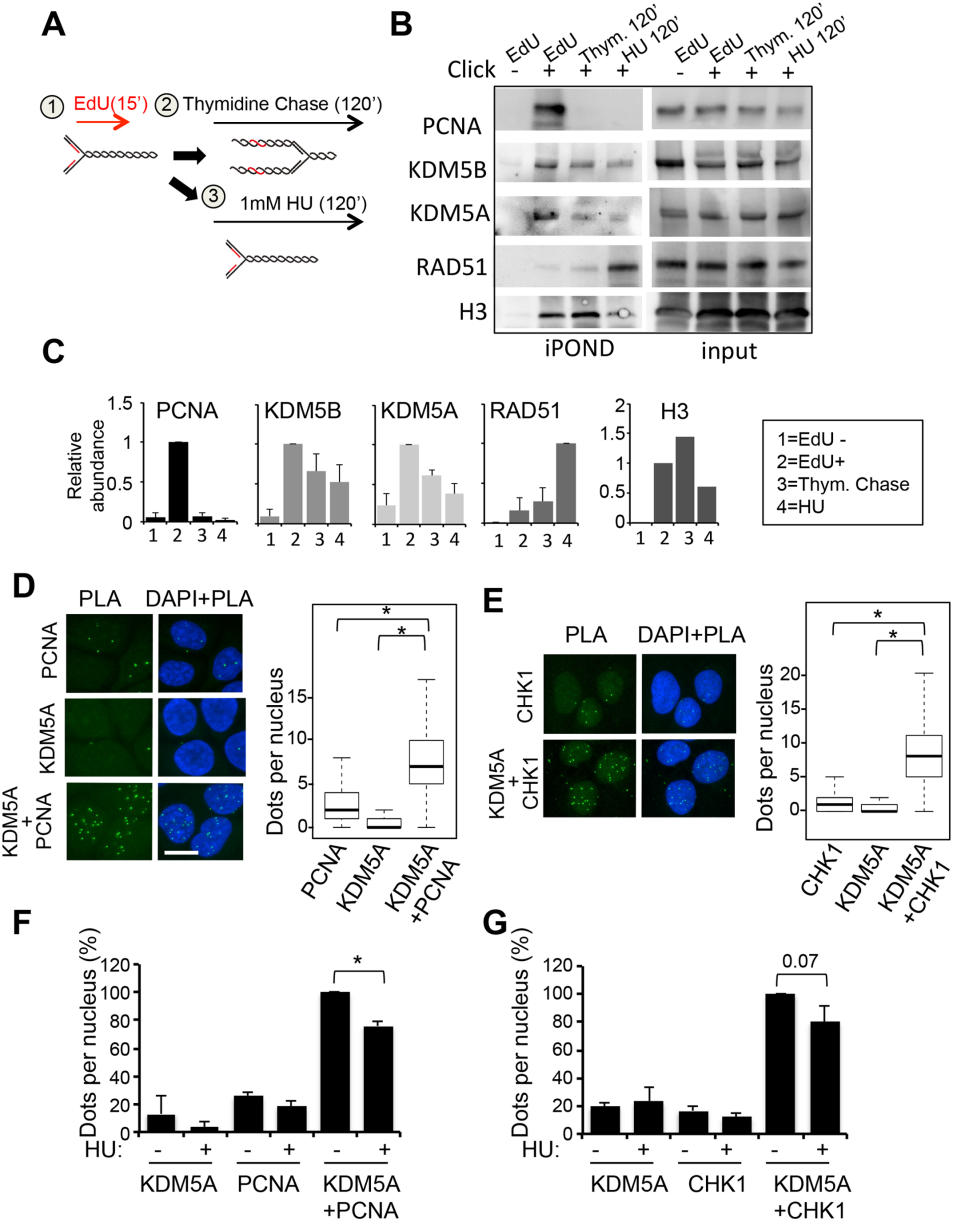
**KDM5A is recruited at replication forks in proximity with PCNA and Chk1.** (A) Schematic description of the iPOND experiment. Active forks are labelled with EdU (1). Upon thymidine chase, labelled DNA is not associated with a fork (2). Upon HU treatment labelled forks are stalled (3). More details can be found in the Materials and Methods section. (B) HeLa S3 cells were labelled with EdU for 15 min (EdU). For thymidine chase experiment, cells were labelled with EdU for 15 min, then washed and 10 mM Thymidine was added for 120 min (Thym 120′). For HU treatment cells were labelled with EdU for 15 min, then washed and 1 mM HU was added for 120 min (HU 120′). EdU labelled DNA fragments were precipitated and co-precipitated proteins analysed by western blot for the presence of PCNA, KDM5A, KDM5B, RAD51 and H3 as a loading control. For the control, the click-it reaction was performed for all samples (+) except for the control (−). (C) Quantification of iPOND experiments. Bar plots indicate the mean and s.e.m. from two independent experiments for PCNA, KDM5B, KDM5A following normalization to 1 for EdU and for RAD51 following normalization to 1 for HU. (D) PLA between KDM5A and PCNA in U2OS cells. Antibodies directed against KDM5A and PCNA were used either separately or together, as indicated. Representative images are shown in the left panel. The number of dots per nucleus was counted in each condition using the Colombus software. Results are presented in the right panel as box-plots showing the median, the 25% and 75% quantiles and extrema below the images, number of counted cells is >100 cells for each point. * indicates a *P*-value <10^−35^. (E) As in D, except that KDM5A antibodies and Chk1 antibodies were used. * indicates a *P*-value<10^−37^. (F) PLA between KDM5A and PCNA as in D, following treatment of cells with 1 mM HU for 1 h (+) or left untreated. The median number of dots was calculated relative to 100 for the sample with the two antibodies together and without HU. *n*=3. mean±s.e.m. *<0.05 (paired *t*-test). (G) PLA between KDM5A and CHK1 as in E, following treatment of cells with 1 mM HU for 1 h (+) or left untreated (−). The median number of dots was calculated relative to 100 for the sample with the two antibodies together and without HU. *n*=3. mean±s.e.m. The *P*-value (paired *t*-test) is indicated.

KDM5C was shown to localize at replication forks and interact with PCNA (Liang et al., 2011; Rondinelli et al., 2015b). We thus tested whether KDM5A and KDM5B could also localize at replication forks. We performed iPOND experiments, which allowed us to analyse the proteins present at on-going replication forks. Cells were labelled 15 min with EdU, which incorporates at active replication forks and allows their purification. As controls, EdU was either not coupled with biotin (Ctl) or EdU-labelled cells were submitted to a thymidine chase in order to compare replication fork versus fork-free chromatin (Fig. 5A). As shown in Fig. 5B and C, KDM5A was specifically enriched at on-going replication forks when compared to the thymidine chase condition, as was PCNA used as a positive control. The specific enrichment of KDM5B was lower. Note that a significant amount of KDM5A and KDM5B persisted following thymidine chase, indicating that they also have a global chromatin distribution in agreement with their role in transcription regulation. Next, we investigated whether KDM5A recruitment at forks is modulated by replication stress, by incubating EdU labelled cells with 1 mM HU. In those conditions, replication is stopped and iPOND allows the isolation of stalled replication forks (Fig. 5A). As previously described (Zellweger et al., 2015), upon HU addition, Rad51 was recruited to stalled forks, whereas PCNA was released (Fig. 5B,C). KDM5A (as well as KDM5B to a lesser extent) behaved as PCNA and was released from the fork, decreasing to the levels observed upon thymidine chase (Fig. 5B,C).

We next investigated whether KDM5A and KDM5B could be in contact with PCNA in cells by performing proximity ligation assays (PLA). Unfortunately, the KDM5B antibody did not give reproducible PLA signals, and we focused on KDM5A. We observed a strong PLA signal only when both the KDM5A and PCNA antibodies were included (Fig. 5D). Importantly, this signal was preferentially detected in S phase cells (Fig. S4) and decreased upon knockdown of KDM5A (Fig. S5), indicating that it is specific for KDM5A. Thus, these data indicate that endogenous KDM5A and PCNA are in close proximity in cells. We next tested the proximity of KDM5A and CHK1, since CHK1 is a known partner of PCNA that localizes at fork in unchallenged conditions (Min et al., 2013; Scorah et al., 2008). We observed a PLA signal when anti-KDM5A and CHK1 antibodies were mixed (Fig. 5E), and this signal was enriched in S phase cells (Fig. S4), and decreased upon depletion of either KDM5A or CHK1 (Fig. S5). Interestingly, the PLA signals between KDM5A and PCNA or CHK1 were diminished upon HU-induced replication stress (Fig. 5F,G). Taken together, these data indicate that in unchallenged conditions KDM5A is recruited to the replication fork in close association with PCNA and CHK1, and that both its recruitment and association with PCNA and CHK1 are regulated by replication stress, opening the possibility that KDM5A participates in the replication process.

### KDM5A/KDM5B-mediated upregulation of RRM2 is crucial for the acquisition of tolerance to replication stress

**Fig. 6.**
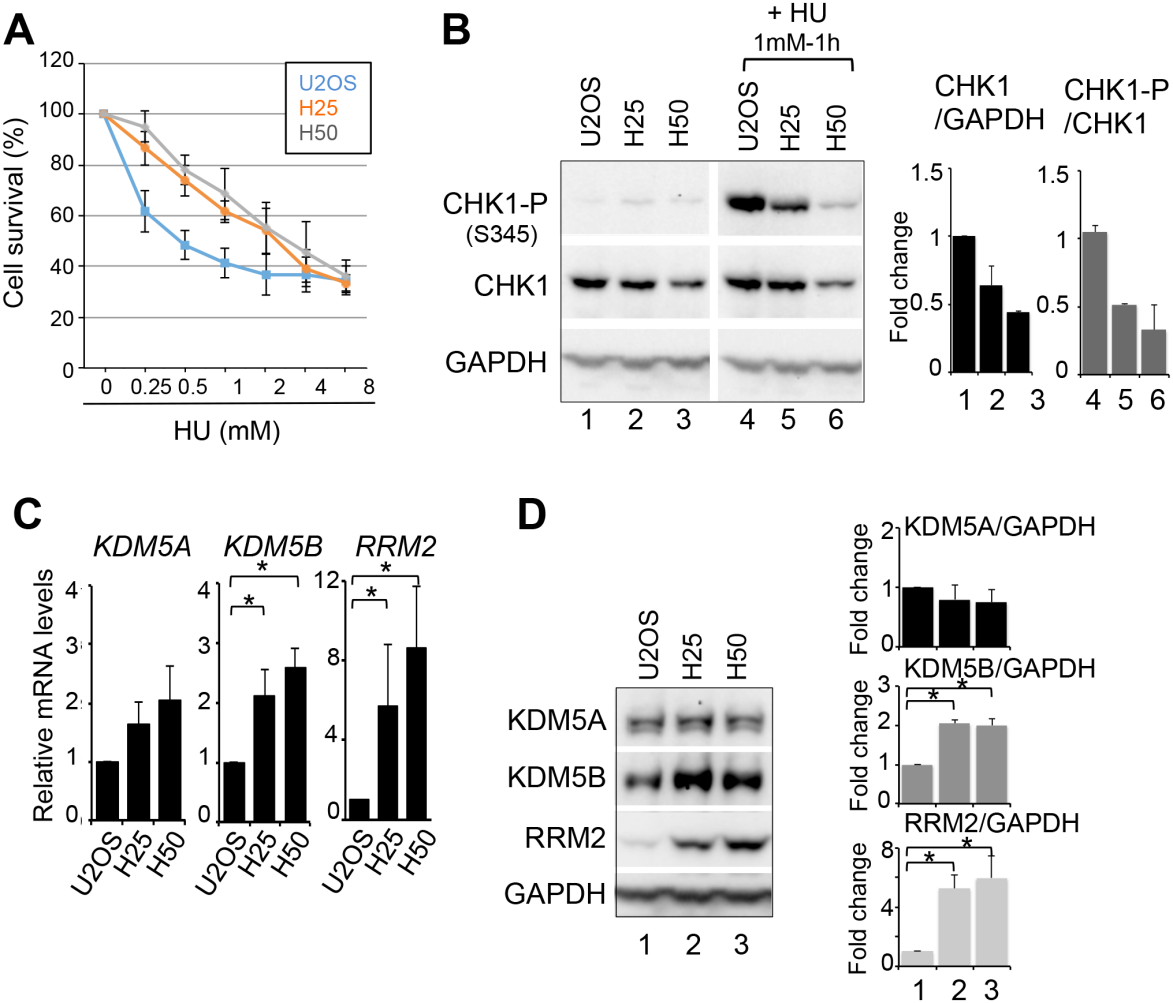
**HU tolerant cells overexpress RRM2 and KDM5B.** (A) Viability of U2OS, H25 and H50, measured by WST assay, 72 h following treatment with increasing doses of HU, as indicated. mean±s.e.m., *n*=3. (B) Left panel: western blot analysis of CHK1 and S345-phospho CHK1 (CHK1-P) expression, in U2OS, H25 and H50 cells before and following 1 h treatment with 1 mM HU. Right panel: quantification following normalization to 1 for U2OS cells. mean±s.e.m., *n*=2. (C) Relative mRNA expression levels of KDM5A, KDM5B, and RRM2 in U2OS, H25 and H50 cells. Expression levels were normalized to the reference gene P0 (ribosomal phosphoprotein P0) and calculated relative to 1 for the siCtl sample. mean±s.d., *n*=3, *P<0.05 (paired *t*-test). (D) Left panel: levels of KDM5A, KDM5B and RRM2 expression levels were analysed by western blot in the parental U2OS cells and its HU tolerant derivatives H25 and H50 grown in 0.25 mM and 0.5 mM HU, respectively. GAPDH is used as a loading control. Right panel: quantification following normalization to 1 for U2OS cells. mean±s.e.m., *n*=3. *P<0.05 (paired *t*-test). For panels B to D, note that H25 and H50 are routinely grown in medium containing 0.25 mM or 0.5 mM, respectively.

KDM5A is a major molecular driver of drug tolerance in cancer cells, allowing the generation of the so-called drug tolerant persisters (DTPs; Sharma et al., 2010; Vinogradova et al., 2016). Resistance of cells to HU depends on the upregulation of RRM2, likely to provide enough dNTPS to support repair of replication stress-induced DNA damages (Choy et al., 1988; Zhang et al., 2009). Because we found that KDM5A and KDM5B are important for the regulation of RRM2 and CHK1 expression, they could be involved in the tolerance of cells to HU. To address this question, we generated U2OS cells resistant to HU by growing cells in 0.25 mM or 0.5 mM HU until they acquired resistance to the drug. Both concentrations led to a block of cells in S phase (data not shown). Cells quickly adapted to the presence of 0.25 mM HU and a population of HU resistant cells was obtained after 10 days of treatment and called H25. Following 0.5 mM HU treatment, a large proportion of cells died and a population of HU-resistant cells was obtained following 1 month, and called H50. Both H25 and H50 showed a marked tolerance to increasing doses of HU (Fig. 6A). This correlated with a decreased expression and lower activation of CHK1 in response to 1 mM HU (Fig. 6B). This lower activation is probably required for these cells to escape the S phase checkpoint. As expected, RRM2 was upregulated several-fold at both the mRNA and protein levels (Fig. 6C,D) in both H25 and H50 cell lines. KDM5A mRNA and protein levels were not significantly changed in both cell lines (Fig. 6C,D). By contrast, KDM5B was more expressed at both the mRNA (Fig. 6C) and protein levels (Fig. 6D). Thus, we conclude that the two cell lines we generated as tolerant to replication stress harboured upregulation of KDM5B and RRM2.

**Fig. 7.**
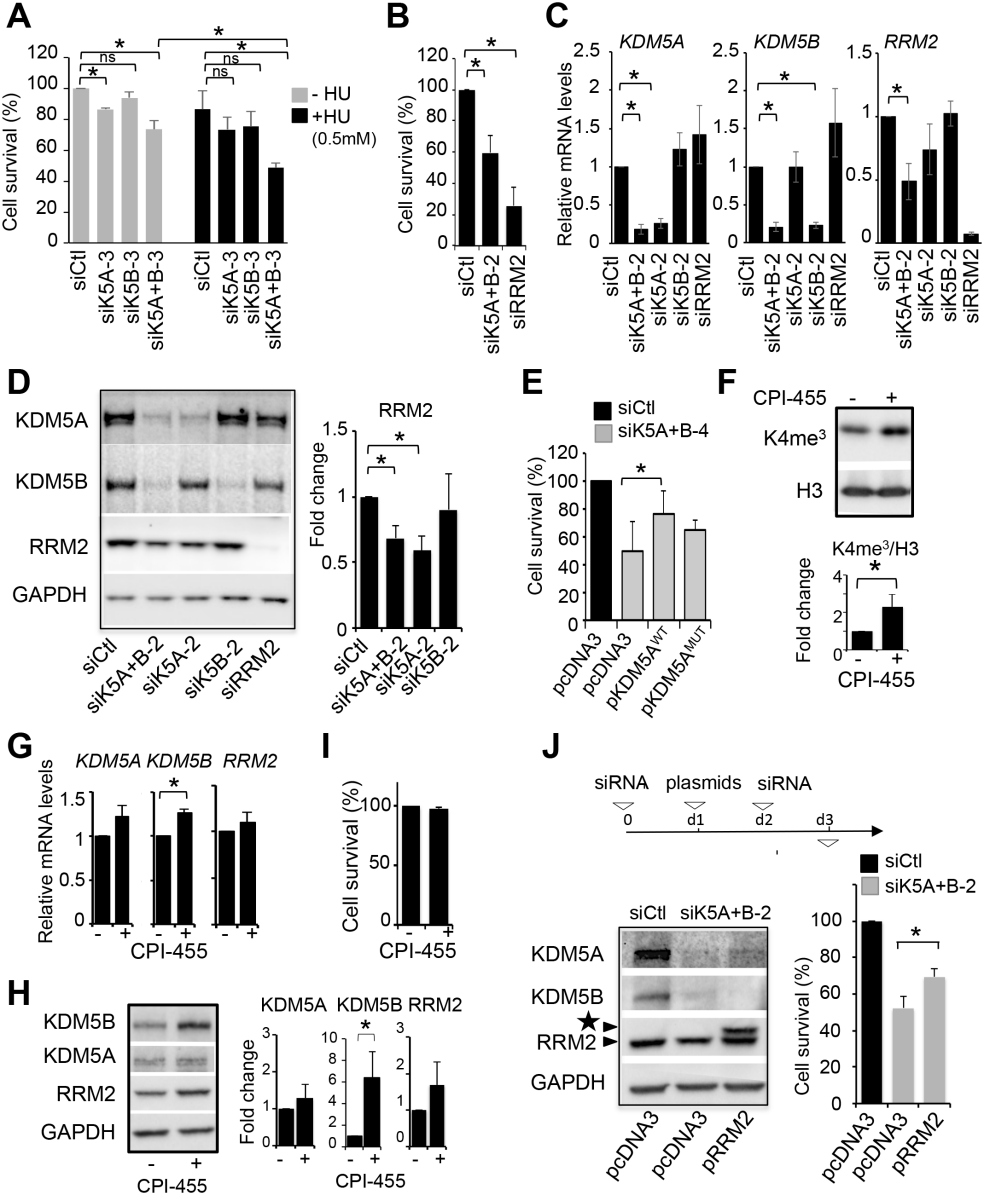
**KDM5A/B are required for replication stress tolerance through regulation of RRM2.** (A) H50 cells grown either in the absence (−HU) or presence of 0.5 mM HU (+HU) were transfected with the indicated siRNA twice in a 48 h interval, and viable cells, excluding Trypan Blue, were counted 96 h after the first transfection. Viability was calculated relative to 100 for si Ctl cells without HU. mean±s.e.m., *n*=3, *P<0.05, ns, non-significant (paired *t*-test). For panels B to I, H50 cells were grown in 0.5 mM HU. (B) Viability of H50 cells grown in 0.5 mM HU upon transfection of the indicated siRNA as described in A, following normalization to 100 for siCtl treated cells. mean±s.e.m, *n*=3. * *P*<0.05 (paired *t*-test). (C) Relative mRNA expression levels of KDM5A, KDM5B, and RRM2, upon transfection of the indicated siRNA in H50 cells. Expression levels were normalized to the reference gene P0 and calculated relative to 1 for the siCtl. mean±s.d., *n*=3. *P<0.05 (paired *t*-test). (D) Western blot analysis of KDM5A, KDM5B, RRM2 and GAPDH as a loading control from H50 cells transfected with the indicated siRNA. Quantification is shown in the right panel with RRM2/GAPDH set to 1 for siCtl. mean±s.e.m., *n*=3. * *P*<0.05 (paired *t*-test). (E) H50 cells were transfected by the indicated siRNA, and re-transfected 24 h later with the indicated expression vector coding for wild-type KDM5A (pKDM5A_WT_) or a histone demethylase-defective mutant (pKDM5A_MUT_). 24 h following plasmids transfection, cells were transfected once more with siRNA. Cells were harvested and counted 24 h after this second siRNA transfection. Bar-plot following normalization to 100 for siCtl+pcDNA3 transfected cells. mean±s.e.m., *n*=3. *P<0.05 (paired *t*-test). (F) Western blot analysis of H3K4me3 and histone H3 from H50 cells treated each 24 h with 12.5 µM KDM5 inhibitor CPI-455 (+) or DMSO (−) for 48 h. A quantification of H3K4me3/H3 is shown on the right with DMSO treated cells set to 1. mean±s.e.m., *n*=3 *P<0.05 (paired *t*-test). (G) Relative mRNA expression levels of KDM5A, KDM5B and RRM2 in H50 cells treated with CPI-455 (+) or DMSO (−) as in F. Expression levels were normalized to the reference gene P0 and calculated relative to 1 for the siCtl sample. mean±s.d., *n*=3. *P<0.05 (paired *t*-test). (H) Western blot analysis of KDM5A, KDM5B, RRM2 and GAPDH from H50 cells treated with CPI-455 (+) or DMSO (−) as in F. Quantification is shown on the right with DMSO treated cells set to 1. mean±s.e.m., *n*=3. *P<0.05 (paired *t*-test). (I) Percentage of living cells following treatment of H50 cells each 24 h with 12.5 µM CPI-455 for 72 h (+) or DMSO (−) following normalization to 100 for DMSO-treated cells. mean±s.e.m., *n*=3. (J) H50 cells were treated with the indicated siRNA (d0) and 24 h (d1) later transfected with pcDNA3-RRM2 (pRRM2) (+) or the empty vector (−). A second siRNA transfection was performed at 48 h (d2) and cells were collected 24 h later (d3). Expression of KDM5A, KDM5B, RRM2 and GAPDH were analysed by western blot (left panel). Note the appearance of a band corresponding to the exogenous tagged RRM2 indicated by a star. Viability was estimated by counting (right panel). siCtl/pcDNA3 transfected cells were set to 100. mean±s.e.m., *n*=4, * *P*<0.05 (paired *t*-test).

We then tested whether this increase in KDM5B expression could be involved in RS tolerance through the upregulation of RRM2. Because the two HU tolerant cell lines behaved similarly, we decided to work with the H50 cell line, resistant to 0.5 mM HU to address this question. First, we questioned whether KDM5A and KDM5B are indeed involved in HU resistance of the H50 cell line derivative. We knocked-down KDM5A and KDM5B either separately or together in H50 cells grown in the absence of HU or maintained in 0.5 mM HU during the time course of experiment. KDM5A and KDM5B depletion affected cell viability in both conditions, but the effect was significantly higher when cells were maintained in 0.5 mM HU (Fig. 7A), showing that KDM5A and KDM5B contribute to HU-resistance. The capacity of H50 cells to grow in the presence of 0.5 mM HU was also reduced using a second couple of siRNA (Fig. 7B).

Upon depletion of KDM5A and KDM5B, the expression of RRM2 was diminished at both the mRNA and protein levels in H50 cells (Fig. 7C,D), similarly to U2OS cells. KDM5A depletion was sufficient by itself to decrease RRM2 expression (Fig. 7D; Fig. S6), suggesting that, in these cells, RRM2 is regulated at the post-transcriptional level by KDM5A but not KDM5B. Importantly, the decrease of cell survival observed upon the concomitant depletion of both KDM5A and KDM5B was rescued, at least in part, by the overexpression of wild-type KDM5A (Fig. 7E), but the effect of the catalytically dead mutant was unclear. To clarify the involvement of their enzymatic activity in this function, we made use of the KDM5 inhibitor CPI-455. As in U2OS cells, treatment of H50 cells with CPI-455 increased in H3K4me3 levels and KDM5B expression (Fig. 7F) but did not affect RRM2 expression at the mRNA or protein levels (Fig. 7G,H). Accordingly, we found no effect on the survival of H50 cells (Fig. 7I). Thus, taken together, these data indicate that KDM5A and KDM5B expression, but not enzymatic activity, is required for the tolerance of H50 cells to replication stress.

We next tested whether this is due to their positive role on RRM2 expression. Strikingly, maintaining RRM2 expression by co-transfecting an expression vector for RRM2 in cells depleted for KDM5A and KDM5B partially restored the viability defect (Fig. 7J), confirming that this defect is due, at least in part, to RRM2 downregulation. Altogether, these results show that the acquired resistance of U2OS cells to HU depends at least in part on the upregulation of RRM2, which is dependent, at least in part, on KDM5A and KDM5B proteins.

## DISCUSSION

In this study, we show that KDM5A and KDM5B proteins are involved in the management of RS induced by HU, by regulating the levels of RRM2, the regulatory subunit of the ribonucleotide reductase RNR, and of CHK1, an effector kinase of RS response. We further demonstrate the importance of the KDM5A/KDM5B-RRM2 axis in the resistance of cells to HU, a potent replication stress inducer. Noticeably, the demethylase-activity of KDM5A and KDM5B is not required for this regulation.

### KDM5A and KDM5B are positive regulators of RRM2

We found that KDM5A and KDM5B act as positive regulators of RRM2, the regulatory subunit of the RNR. RNR is a tetrameric enzyme formed by the association of two RRM1 large subunits and two RRM2 small subunits. RRM2, but not RRM1, is regulated in a cell cycle dependent manner. Noticeably, KDM5A and KDM5B have redundant roles for RRM2 regulation at the transcriptional level. Indeed, a significant effect is only seen when depleting both proteins, indicating that the presence of one protein compensates for the absence of the other. Note, however, that depleting both KDM5A and KDM5B together has only a twofold effect on RRM2 expression. It is thus possible that other members of the KDM5 family, in particular KDM5C, also participate in RRM2 and compensate to some extent the absence of KDM5A and KDM5B. Depleting KDM5A is sufficient by itself to decrease RRM2 protein expression in H50 cells, suggesting that it also contributes to regulate RRM2 at the post-transcriptional level.

As enzymes removing the H3K4me3 mark present at active genes promoters, KDM5A and KDM5B are often described as transcriptional repressors. Positive roles of KDM5 family members on gene expression have however been described. For example, association of KDM5A and KDM5B to the MRG15 complex was proposed to favour the elongation step of transcription by demethylating H3K4me3 in the body of some genes (Hayakawa et al., 2007). Here, we show that KDM5A is found at the promoter of RRM2, but not in the coding sequence excluding such mechanism. Our attempts to perform KDM5B ChIP were unsuccessful and we cannot exclude that KDM5B could be present on the coding region of *RRM2*. Another possibility would be that KDM5A or KDM5B modulates RRM2 enhancer function, since KDM5C and KDM5B itself were already shown to regulate enhancer activity (Kidder et al., 2014; Outchkourov et al., 2013; Shen et al., 2016).

The above-described mechanisms depend on H3K4me3 removal and thus on the enzymatic activity of KDM5 proteins. We found that KDM5A and KDM5B regulate RRM2 in a demethylase-independent manner. Some examples in which KDM5A and KDM5B regulate gene expression or cellular processes independently of their enzymatic activity are already described. In *Drosophila*, Lid, which is the only expressed KDM5 family member, is crucial for larval growth in a manner that is independent of the JMJc domain carrying demethylase activity (Drelon et al., 2018). Lid functions as a positive regulator of mitochondrial genes, in a manner that depends on its PHD3 domain. This domain allows the recruitment of Lid to chromatin by binding to H3K4me3, and may interact with transcriptional co-activators that remain to be identified (Liu and Secombe, 2015).

KDM5A and KDM5B could similarly recruit transcriptional co-activators to the RRM2 promoter. Such a co-activator could be Tip60, a histone acetyl transferase contained in the MRG15 complex. Equally possible is that KDM5A could impede the access to the promoter or the activity towards chromatin of a co-repressor. Clearly, how KDM5A/KDM5B positively regulates RRM2, in a demethylase-independent manner merits further investigations.

### KDM5A localizes at forks and interacts with PCNA and CHK1

In this manuscript, we observed that KDM5A is enriched at active replication forks in close association with PCNA and CHK1. The ability of KDM5A to interact with PCNA is shared with KDM5C, which contacts PCNA through a PIP box of sequence QCDLCQDWF (Liang et al., 2011). Interestingly, this sequence is conserved in KDM5A, but not in KDM5B, and likely mediates the binding of KDM5A to PCNA. KDM5C was shown to regulate replication by dictating the binding of crucial players implicated in origin firing and DNA polymerase processing such as CDC45 and PCNA (Rondinelli et al., 2015b). We found that depletion of KDM5A alone, but not KDM5B diminishes EdU incorporation in S phase cells treated or not with HU, although treatment with HU increases this effect. This suggests that KDM5A may have a specific function in replication, which cannot be compensated by KDM5C. What is the function of KDM5A at the fork in association with PCNA is unclear for the moment. However, the fact that both its localization at the fork and its proximity with PCNA are regulated by HU suggests its involvement in replication stress response.

Given that it also localizes at close proximity to CHK1, one could speculate that it is involved in the CHK1-dependent signalling. Our data argue, however, against a direct function of KDM5A in regulation the ATR/CHK1 pathway, since the effect of KDM5A and KDM5B on CHK1 is mainly due to the regulation of its expression.

### KDM5A and KDM5B and replication stress tolerance

KDM5A is involved in the emergence of the so-called DTPs in cancer (Sharma et al., 2010; Vinogradova et al., 2016). KDM5A upregulation allows the emergence of cells tolerant to cisplatin, a DNA damaging agent (Haven et al., 2016; Vinogradova et al., 2016). In contrast, we show that KDM5A levels do not increase in cells becoming resistant to the replication stress-inducer HU. However, KDM5B is upregulated when compared to parental U2OS. Interestingly, KDM5B overexpression has been associated with chemotherapy resistance in epithelial ovarian cancer (Wang et al., 2015), with the development of Glioma (Fang et al., 2016), and with the presence of a subpopulation of slow-cycling cells involved in long-term tumour maintenance in melanoma (Roesch et al., 2010). Thus, either KDM5A or KDM5B could fulfil the same function in cancer development/drug resistance, depending on the cell context. Accordingly, we show that KDM5A and KDM5B act redundantly in the regulation of RRM2 gene expression, meaning that they can compensate each other. This observation is in line with other studies describing a redundant role of KDM5 family members on gene expression (Brier et al., 2017; Chicas et al., 2012; Islam et al., 2011).

#### What is the mechanism by which KDM5A and KDM5B contributes to HU resistance?

Although KDM5A and KDM5B are important to regulate CHK1 expression in U2OS cells in response to HU, this does not seems to contribute to the resistance phenotype of H50 cells. Indeed, in these cells, CHK1 activation is very compromised. This decrease probably participates in allowing H50 cells to escape the intra S checkpoint despite the continuous replicative stress that they face. Given that the ATR-CHK1 pathway promotes RRM2 accumulation in cycling cells (Buisson et al., 2015), one could expect RRM2 levels to follow that of CHK1. However, in contrast, we observe an upregulation of RRM2. Although RRM2 expression in H50 cells is decreased upon depletion of KDM5A and KDM5B, it is not back to its levels in parental U2OS cells. This indicates that, even upon KDM5A/KDM5B depletion, the coupling between CHK1 and RRM2 is not restored. The most likely explanation is that other mechanisms are involved in RRM2 upregulation in these cells. For example, XRCC2, a RAD51 paralog, negatively regulates RRM2 through an unknown mechanism (Saxena et al., 2018). Interestingly, XRRC2 is regulated by ATR independently of CHK1. This mechanisms or yet undescribed mechanisms could be responsible for the uncoupling between CHK1 and RRM2 expression.

Our data suggest that the KDM5A and KDM5B-dependent tolerance of H50 cells probably relies, at least in part, on the other mechanism we describe in our study, i.e. RRM2 expression control. This would fit with our finding that RRM2 is overexpressed in H50 cells and with the widely described function of RRM2 in allowing cells to cope with replicative stress. It is, however, puzzling that although KDM5B expression increases in HU-resistant cells, the expression of RRM2 is affected by the depletion of KDM5A alone. We can speculate that KDM5B has some specific and important functions in HU-resistant cells, so that the correct expression of RRM2 relies exclusively on KDM5A. In agreement with this hypothesis, overexpression of RRM2 is not sufficient to fully complement the defect observed upon KDM5A and KDM5B depletion, consistent with the existence of yet unidentified mechanisms.

Strikingly, although KDM5A and KDM5B expression are important for HU resistance, we report here that their histone demethylase activity is not essential. This stands in contrast to what has been previously described for tolerance to other drugs (Sharma et al., 2010; Vinogradova et al., 2016). One explanation of this difference could be that previous reports addressed a role of KDM5A in the initiation of drug tolerance and not on its maintenance, which is clearly the step we study using the H50 model. Alternatively, the mechanisms involved may be drug-specific with specific mechanisms taking place to achieve tolerance to replication stress. As previously discussed, KDM5A and KDM5B may recruit to chromatin distinct chromatin regulators and/or inhibit the activity of negative regulators such as HDAC and as such may regulate nuclear events independently of their demethylase activity (DiTacchio et al., 2011; Liu and Secombe, 2015). Nevertheless, our study reveals the importance of assessing the requirement of the demethylase activity in KDM5 proteins oncogenic functions, which may depend on cell types and cancer stages, as well as on the chemotherapeutic used to treat cancer. Our study also underlines the importance of designing new allosteric inhibitors of KDM5A and KDM5B, which impede partners association instead of inhibiting demethylase activity.

## MATERIALS AND METHODS

### Cell culture

U2OS and HeLa S3 cells were obtained from ATCC and cultured in Dulbecco's modified Eagle's medium (DMEM-5.5 g/l glucose) plus 10% FBS. Medium was supplemented with 100 U/ml penicillin, 100 µg/ml of streptomycin (Gibco) and 1 mM of Sodium Pyruvate (only for U2OS). H25 and H50 cells were established from U2OS cell line, by adding HU at a concentration of 0.25 or 0.5 mM in the medium. HU was changed every 2 days until they became tolerant to the drug. Cells were grown in the presence of HU used at the concentration of selection for all experiments. Cells were maintained in a 37°C incubator with 5% CO2.

### Antibodies

The following antibodies were used: anti-KDM5A (D28B10-Cell Signaling Technology), anti-KDM5B (CL1147-Thermo Fisher Scientific), anti-RRM2 (2G1D5-Cell Signaling Technology), anti-CHK1 (2G1D5-Cell Signaling Technology for western blot; C9358-Sigma-Aldrich for PLA), anti-CHK1 phospho-Ser345 (133D3-Cell Signaling Technology), anti-ATR (E1S3S-Cell Signaling Technology), anti-ATR phospho-Thr1989 (GTX128145, GeneTex), anti-RPA (Subunit 9H8) (Santa Cruz Biotechnology SC56770), anti-RPA phospho-Ser33 (A300-246A-T- Bethyl), anti-PCNA (CBL407-Millipore), anti-RAD51 (SC8349-Santa Cruz Biotechnology), anti-BRCA1(SC642-Santa Cruz Biotechnology), anti-H3K4Me3 (12209-Abcam), anti-H3 (1791-Abcam), anti-GAPDH (MAB374-Millipore), anti-γH2AX (Ser139) (20E3-Cell Signaling Technology or JBW301, Millipore), anti-53BP1 (NB100-304-Novus).

### Transfection/electroporation

2.10^6^ cells were electroporated with double-stranded siRNA to a final concentration of 2 µM using an electroporation device (Lonza 4D Nucleofector) with the SE cell line 4D nucleofector kit L according to the manufacturer's specifications. Alternatively, siRNAs were transfected with interferin at a final concentration of 20 nM following the manufacturer's instructions. The following siRNA were used: siGENOME non-targeting control smartpool #1 and #2 from Dharmacon (Horizon-Discovery) were used as control. siKDM5A-1 and siKDM5B-1 were siGENOME smart pool purchased from Dharmacon (Horizon Discovery). Other siRNAs were purchased from Eurogentec including:

siKDM5A-2:5′-GGAUGAACAUUCUGCCGAAdTdT-3′,

siKDM5B-2,5′-GGAGAUGCACUUCGAUAUAdTdT-3′,

siKDM5A-3:5′-UGACAAUGGUGGACCGCAUdTdT-3′,

siKDM5B-3:5′-CCACAGAGCUUGUUGAGAAdTdT-3′;

siKDM5A-4:5′-UAAGCCUCUAACUACUAUCAGdTdT3′,

siRRM2: 5′-UGAACUUCUUGGCUAAAUCUUdTdT-3′.

Total siRNAs amounts were kept identical between each point using a mix 1:1 of control siRNA-1 and −2, a mix 1:1 of either siKDM5A or siKDM5B or siRRM2 siRNA and control siRNA-2, or a mix 1:1 of siKDM5A and siKDM5B.

Transfection of plasmids was done using the U20S-Avalanche reagent (Cambio), as described by the supplier. The plasmid pcDNA3-RRM2 (indicated as pRRM2 in Fig. 7) was purchased from Addgene. Plasmids coding for wild-type KDM5A (pcDNA3/HA-FLAG KDM5A) or a demethylase dead mutant (pcDNA3/HA-FLAG KDM5A H483A) are a gift from W. Kaelin. Empty pCDNA3 was used as control. For rescue experiments, cells were first electroporated with siRNA and 24 h later transfected with plasmids. siRNA (20 nM) were transfected a second time at 48 h using the Interferin^™^ polyplus reagent (Ozyme). Cells were collected at 72 h.

### Cell viability and clonogenic assay

Cell viability was estimated using the WST assay (Sigma-Aldrich). For this purpose, cells were plated in 96 wells plate. After 24 h, cells were incubated with or without HU (Sigma-Aldrich) or CPI-455 (12.5 µM, Selleckchem). After 72 h of treatment, WST-1[2-(4-Iodophenyl)-3-(4-nitrophenyl)-5-(2,4-disulfophenyl)-2H-tetrazolium] was added to the medium at a dilution of 1/10, followed by an incubation at 37°C for 2 h, before measuring the absorbance at 450 nm. Alternatively, cells were mixed with Trypan Blue and counted using the countess II automated cell counter (Life Technologies).

For clonogenic assay, U2OS cells were electroporated with siRNA as described before and seeded at 30 cells/cm2 in triplicates in six-well dishes. The day after, they were treated for 24 h with 50 µM HU or left untreated and then were allowed to grow for 10–15 days more before fixation and coloration with 1% Crystal Violet in H_2_O.

### Western blotting

Cells were lysed with Lysis buffer S (20 mM Tris-HCl pH 7.2, 1% SDS) for most experiments. For detection of ATR and ATR-P they were lyzed in buffer N (20 mM Tris-HCl pH 8, 0.4% NP40, 300 mM NaCl, 1 mM DTT). Total protein concentration was measured using the Bio-Rad protein assay kit following the manufacturer's instructions. 20–50 µg were loaded per well of a 3–8% Tris-Acetate gradient minigel (NuPage), or a 4–12% Bis-Tris for small proteins like H3 and RPA. Western blots were performed using standard procedures. Antibodies used are listed in the paragraph ‘antibodies’. HRP-conjugated secondary antibodies were purchased from Amersham and Bio-Rad. Western blots were revealed by ECL (Bio-Rad). Images were acquired using the Chemidoc touch imaging system from Bio-Rad. Western blots were quantified with ImageJ.

### Total RNA extraction and RT-qPCR

RNA was extracted using an RNeasy mini kit (Qiagen) as described by the supplier. 500 ng of purified RNA were reverse-transcribed by the PromII reverse transcriptase (Promega) using 0.5 µg of random primers following the supplier's protocol. cDNAs were analysed by q-PCR on a CFX96 real-time system device (Bio-Rad) using the platinum SYBR Green qPCR SuperMix (Invitrogen), according to the manufacturer's instructions. All experiments included a standard curve.

The primers used were P0 forward : 5′-GCGACCTGGAAGTCCAACT-3′ and reverse 5′-CCATCAGCACCACAGCCTTC-3′; KDM5A forward 5′-TGAACGATGGGAAGAA AAGG-3′ and reverse 5′-AGCGTAATTGCTGCCACTCT-3′; KDM5B forward 5′-GAGCTGTTGCCAGATGATGA-3′ and reverse 5′-TGATGCAGGCAAACAAGAAG-3′; RRM2 forward 5′-TTCTTTGCAGCAAGCGATGG-3′ and reverse 5′-TTCTTTGCAG CAAGCGATGG-3′; CLASPIN forward 5′-TAAACCACGGCTAGGTGCTG-3′ and reverse 5′-AGGCTTCCAGTTCTCTGTTGG-3′; TOPBP1 forward 5′-AGCCCTCAACTG AAAGAGGC-3′ and reverse 5′-AACTCCACCTGTAATCTGCTCC-3′; RAD9 forward 5′-CTTCTCTCCTGCACTGGCTG-3′ and reverse 5′-CTTTGGCAGTGCTGTCTGC-3′; RAD1 forward 5′-CAGGGACTTTGCTGAGAAGG-3′ and reverse 5′-GGCCACAAGGCT GTACTGAT-3′; MDC1 forward 5′-TCCGACGGACCAAACTTAAC-3′ and reverse 5′-ATCAGTGACCAGGTGGGAAG-3′; HUS1 forward 5′-CAGAAACGTGGAACACATGG-3′ and reverse 5′-ACAGCGCAGGGATGAAATAC-3′; CHK1 forward 5′-AGAAA GCCGGAAGTCAACAC-3′ and reverse 5′-AGACTTGTGAGAAGTTGGGCT-3′; ATR forward 5′-ACATTTGTGACTGGAGTAGAAGA-3′ and reverse 5′-TCCACAATTGGTG ACCTGGG-3′; CDC6 forward 5′-GCAAGAAGGCACTTGCTACC-3′ and reverse 5′-GCAGGCAGTTTTTCCAGTTC-3′; CCNE1 forward 5′-AGGGGACTTAAACGCCACTT-3′ and reverse 5′-CCTCCAAAGTTGCACCAGTT-3′.

### High-throughput microscopy

The Operetta automated high-content screening microscope (PerkinElmer) was used for quantification of γH2AX, 53BP1 bodies and/or cell cycle analyses. Cells seeded on a 96-well plate were fixed with 4% of freshly prepared paraformaldehyde and permeabilized with 1% Triton X-100 in PBS. For 53BP1 staining, cells were permeabilized with 1%Triton X100 in PBS, on ice for 5 min, prior to fixation. A blocking step was performed with 1%BSA for 30 min at room temperature. Cells were then incubated with primary antibodies in PBS-1%BSA overnight at 4°C. After three washes, cells were incubated with secondary antibodies (goat anti-rabbit Alexa 647 and/or donkey anti-mouse Alexa 488) at a 1/1000 dilution in PBS-1%BSA for 2 h at room temperature. After three washes, a DAPI staining was performed for 10 min. For labelling S phase, cells were labelled with EdU for 20 min prior to fixation. EdU was revealed before incubation with primary antibodies, using the click-it imaging kit (Thermo Fisher Scientific) following the supplier's instructions. Antibodies are described in the paragraph ‘antibodies’. Image acquisition with a 20× objective lens was automated to obtain at least 20 fields per well, allowing the visualization of a total of 500–1000 cells (three wells were acquired for each condition). Each picture was analysed with the integrated Columbus software. Briefly, the DAPI-stained nuclei were selected (method B), and when necessary the size and roundness of nuclei were used as parameters to eliminate false positive compounds. For cells treated 24 h with 50 µM HU, the γ-H2AX staining was delineated using the find spot methods A or B and the sum intensity of the spots was measured. For cells treated with 1 mM for up to 60 min, the sum intensity of γ-H2AX per nucleus was measured. For cell cycle analysis, the sum of the DAPI intensity and the mean of the EdU intensity were plotted in order to separate G1, S, and G2 cells. The sum of the γH2AX intensity was subsequently determined in each of these cell population. When EdU labelling was not possible cells were separated in G1, S, and G2 phases according to DAPI sum staining as described in Roukos et al. (2015). 53BP1 bodies were delineated using the find spot method B. As a control, cells were treated with 0.2 µM aphidicolin for 24 h. Box-and-whisker plots of quantification of γH2AX staining were obtained with the R open source software R Core Team version 3.5.2 (2018-12-20; http://www.R-project.org/). They show the median, the 25 and 75% quantiles. Outliers, even if they are not shown are not excluded from the computations and tests [outliers are identified by not being in the range (25%Quantile−1.5×InterQuantiles; 75%Quantile+1.5×InterQuantiles) where interQuantiles=75%Quantile−25%Quantile]. These representations have to be accompanied by statistical analysis of the comparison between the two populations. Statistical hypothesis tests were applied to confirm whether the hypothesis (that can be seen on the boxplot) that there is a differences between indicators of the two populations (such as mean, median, distribution) can be considered as true with a great confidence or can be due to random effect. Because data distribution was not normal (normality tested with Shapiro–Wilk test), we used a Wilcoxon test to reject the hypothesis that the two populations medians are the same and thus conclude that there is a significant difference between the two medians if the *P-*value is <0.05, meaning a confidence of 95%.

### iPOND

We isolated proteins on nascent DNA (iPOND) as described previously (Lossaint et al., 2013; Sirbu et al., 2011). Newly synthesized DNA in Hela S3 cells (∼2.5.10^8^ per experiment) was labelled by incubation with 10 µM EdU for 15 min. For pulse-chase experiments with thymidine (Sigma-Aldrich), cells were washed with cell culture medium supplemented with 10 µM thymidine, and incubated for 120 min. in thymidine-containing medium. For HU treatment, cells were washed and placed into medium containing 1 mM HU for 120 min. Then the cells were cross-linked with 2% formaldehyde for 15 min. For the conjugation of EdU with biotin TEG azide, cells were permeabilized with 0.5% triton X-100, washed with 1×PBS, and then incubated for 2 h in Click reaction buffer [10 mM Sodium-L-Ascorbate, 10 mM biotin TEG Azide (Glenresearch), 2 mM CuSO4]. Cell pellets were washed with PBS, and then resuspended in lysis buffer (10 mM Hepes-NaOH, 100 mM NaCl, 2 mM EDTA, 1 mM EGTA, 1 mM PMSF, 0.2% SDS, 0.1% sarkozyl, Roche proteases inhibitor). Sonication was performed with a Misonix sonicator (15 cycles of 20 s sonication interspaced by a pause of 50 s). For the isolation of proteins on EdU-labelled DNA, samples were centrifuged 10 min at 18,000× ***g*** and supernatants were incubated overnight with streptavidin-coupled magnetic beads from (Ademtech). An aliquot (2%) of the extract was kept as loading control. To reverse crosslinks and recover proteins bound to magnetic beads, the beads were washed in lysis buffer and then incubated in Laemmli buffer for 30 min at 95°C with shaking.

### PLA

The *in situ* PLA was performed with DuoLink PLA technology probes and reagents (Sigma-Aldrich). Cells were fixed and processed as described above for immunofluorescence, except that the secondary antibodies were those provided with the PLA kit. Antibodies used are described in the paragraph ‘antibodies’. Revelation was performed according to the supplier’s instructions. Images were acquired with a fluorescence microscope (DM500, Leica) coupled to Metamorph and analysed using the Colombus program.

### Chromatin immunoprecipitation

Cells were grown until 80% confluence and cross-linked with 2% formaldehyde for 10 min before addition of 0.125 M glycine for 5 min. Fixed cells were washed with PBS and harvested by scrapping. Pelleted cells were lysed with the following buffer: Pipes 5 mM pH 8, KCl 85 mM, NP-40 at 0.5%. The lysis was followed by homogenisation with a Dounce homogeniser. Nuclei were harvested by centrifugation and incubated in a nuclear lysis buffer: 50 mM Tris pH 8.1, 10 mM EDTA, 1% SDS. Samples were diluted ten times in a dilution buffer: 0.01% SDS, 1.1% Triton X-100, 1.2 mM EDTA pH8, 16 mM Tris pH8.1, 167 mM NaCl. A sonication step was performed ten times for 10 s at a power setting of 5 and a duty cycle of 50% (Branson Sonifier 250) to obtain DNA fragments of about 500–1000 bp. A preclearing step was made for 2 h at 4°C with 50 µl of previously blocked protein-A and protein-G beads (Sigma-Aldrich) for 200 µg of chromatin. Beads blocking was achieved by incubating the agarose beads with 200 µg/ml of herring sperm DNA and 500 µg/ml of BSA for 3 h at 4°C. After preclearing, samples were incubated with antibodies specific for KDM5A (1 γg/ml) or without antibody as negative control overnight at 4°C. Then, 50 µl of blocked beads were added to the immune complexes for 2 h at 4°C on a rotating wheel. Beads were washed once in dialysis buffer (2 mM EDTA, 50 mM Tris pH8, 0.2% Sarkosyl) and five times in wash buffer (100 mM Tris pH 8.8, 500 mM LiCl, 1% NP40, 1% NaDoc). Elution from beads was achieved by incubation in elution buffer (1%SDS, 100 mM NaHCO3). Crosslinking was reversed by adding to samples RNase A (10 mg/ml) for 30 min at 37°C and incubating with 4 µl SDS 10% overnight at 70°C. After 2 h of proteinase K treatment, DNA was purified on a GFX column (GFX PCR kit, Amersham) and analysed by q-PCR.

The primers used were: CDC6-P forward 5′-CAGTTTGTTCAGGGGCTTGT-3′ and reverse 5′-GCTCAGCTCTTTTCCCTTCA-3′; CDC6 coding : forward 5′-TGCTAATACCCT GGATCTCACA-3′ and reverse 5′-CTGATTTCTGGTATAAGGTGGGA-3′; RRM2-P forward 5′-CTCAGCGGCCCTAACTTT-3′ and reverse 5′-CTTTCGATCCGTGTCCCT-3′; RRM2-coding forward 5′-AAAGCCAGGAGCATGAACTC-3′ and reverse 5′-TCCCAATCCAGTAAGGAAGG-3′; CHK1-P forward 5′-CATCTCCACGTCACCCTTTT-3′ and reverse 5′-ACCACTGCAGGAATCCAAAT-3′; CHK1-coding forward 5′-GCGAT TATTGCCACCCTAAA-3′ and reverse 5′-GGGTTTAAGCATTGCGGTTA-3′; MYOG-P forward 5′-GAATCACATCTAATCCACTCTA-3′and reverse 5′-ACGCCAACT GCTGGGTGCA-3′.

### Statistical analyses

Unless specified in the figure legend, statistical analyses for qPCR, western blot and viability data were performed using student paired *t*-test. The Wilcoxon test was used for the analysis of fluorescence data. Unless specified, the star * indicates a *P*-value<0.05. Either ns or nothing on the graph indicates that the difference to the control was not significant.

## Supplementary Material

Supplementary information
