# Supplementary material for: KDM5A and KDM5B histone-demethylases contribute to HU-induced replication stress response and tolerance
Source: Biol Open. 2021 May 26;10(5):bio057729. doi: 10.1242/bio.057729 (PMC8181900; doi:10.1242/bio.057729)
Supplement: Supplementary information [file biolopen-10-057729-s1.pdf]

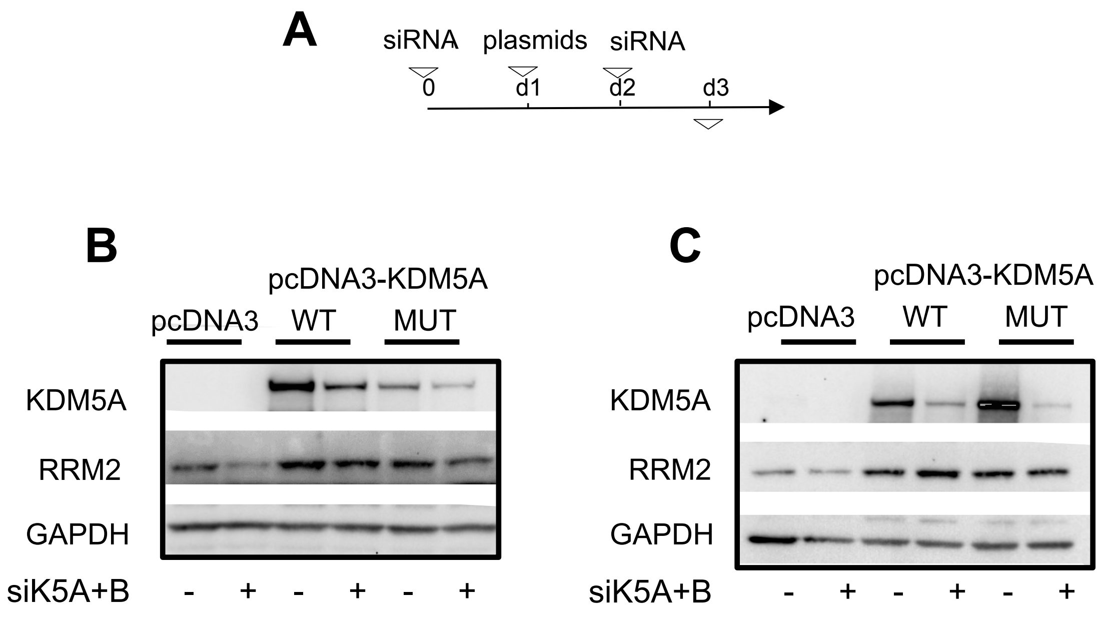

Figure S1. Western-blot analysis of the rescue experiment presented in Fig 3B. **A**-U2OS cells were electroporated (d0) with siRNA targeting KDM5A (siK5A-4) and KDM5B (siK5A-2) and transfected 24 hours (d1) later with expression vectors either empty (pcDNA3) or coding for KDM5A either wild-type (WT) or a histone demethylase-defective mutant (MUT). To ensure efficient knockdown of KDM5A and KDM5B, cells were transfected once more with siRNA 24 hours following plasmids transfection (d2). Cells were collected 24 hours after this second transfection (d3), counted (figure 3B) and processed for western-blot analysis of KDM5A (using the anti-KDM5A antibody), RRM2 and GAPDH as a loading control. Two independent experiments (**B** and **C**) are shown.

Biology Open • Supplementary information

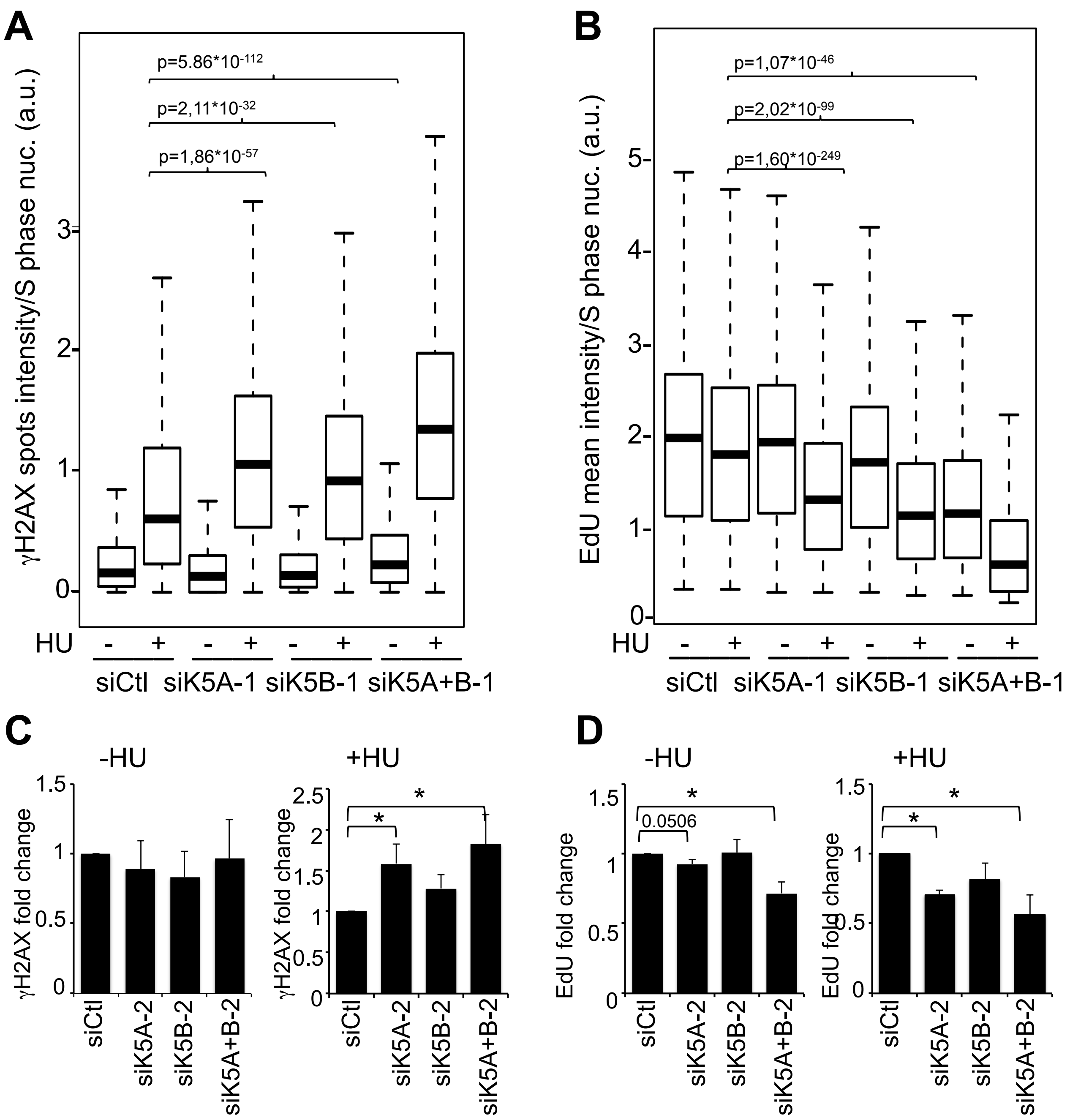

Figure S2. Replication stress increases upon depletion of KDM5A or/and KDM5B: A-B: U2OS cells were treated and analyzed as in figure 3D-F using a distinct couple of siRNA (siK5A+B-1). A non-targeting siRNA pool was used as control (siCtl). KDM5A or/and KDM5B depleted cells were incubated with 50 μM HU during 24 hours (+) or left untreated(-). Cells were labeled with EdU before fixation and stained for EdU, DAPI and γ-H2AX, allowing to quantify γ-H2AX spots intensity (**A**) and EdU staining (**B**) in S phase nuclei. Results are represented as box-plots. A representative experiment out of 2 is shown. Number of quantified S-phase cells was >1000 for each point. pvalues are indicated on the graph. **C-** Fold change of the median of γH2AX spots intensity in siKDM5A-2 or/ and siKDM5B-2 treated cells relative to control without (-HU) or following HU treatment (+HU). n=3. * $p < 0.05$ (paired t-test). **D-** Fold change of the median of EdU intensity in siKDM5A-2 or/and siKDM5B-2 treated cells relative to control without (-HU) or following HU treatment (+HU). n=3. * $p < 0.05$, p=0.0506 between siK5A-2 and siCtl cells, in HU untreated condition (paired t-test).

Biology Open • Supplementary information

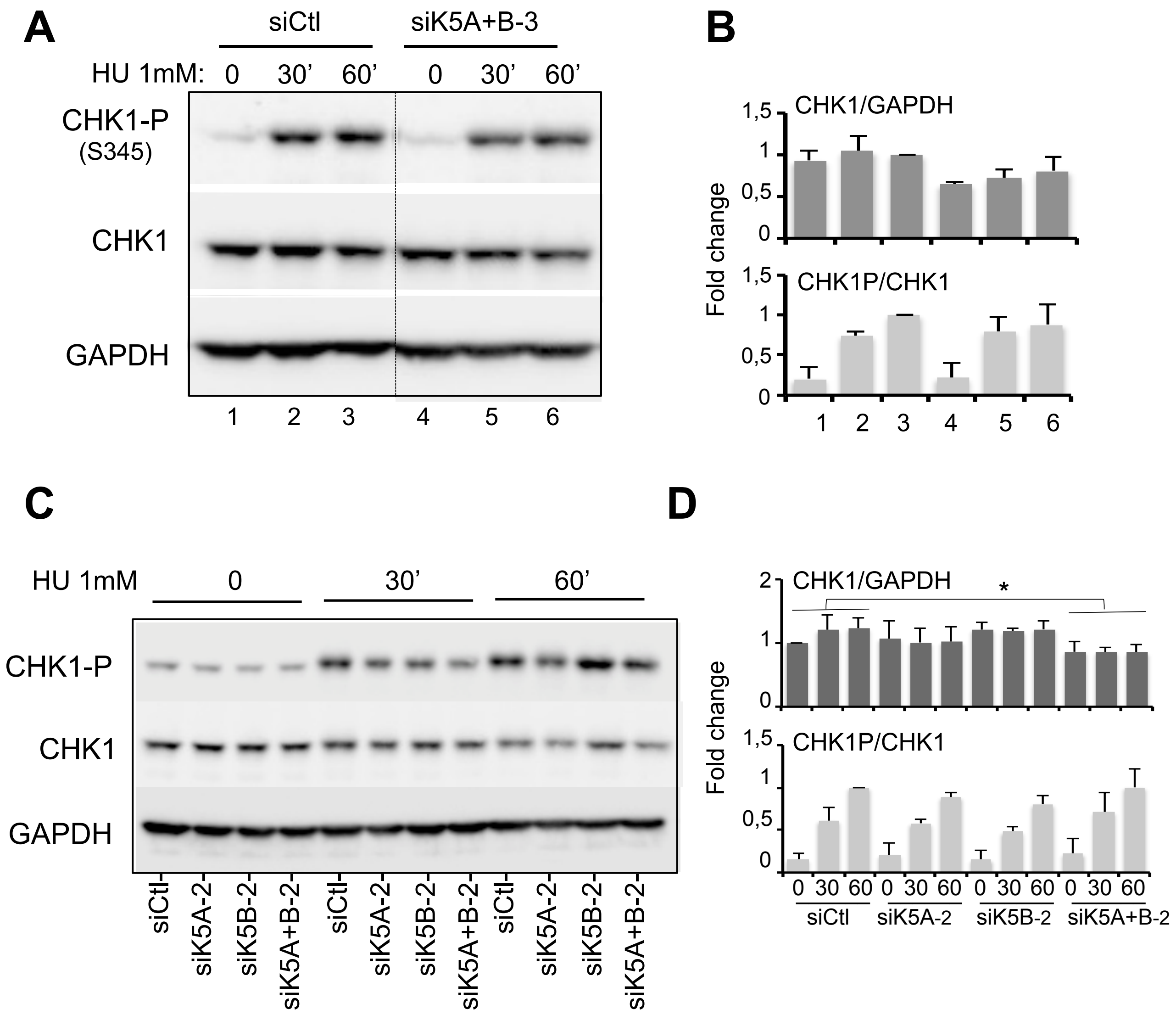

Figure S3. KDM5A and KDM5B depletion decreases the total amount of CHK1

A-U2OS cells were electroporated with siRNA against KDM5A and KDM5B (siK5A+B-3) or a non-targeting pool of siRNA (siCtl). 48h later, cells were treated with 1 mM HU during30 and 60 min as indicated, and processed for western-blot analysis of CHK1, S345-phosphorylated CHK1 (CHK1-P) and GAPDH**.** **B**- Quantification of A with CHK1 normalized to GAPDH and CHK1-P normalized to CHK1. mean +/- s.e.m. n=2. **C**- U2OS were treated as in Fig4B. siK5A-2 and siK5B-2 were transfected either individually or together. **D-** Quantification of C, with CHK1 normalized to GAPDH and CHK1-P normalized to CHK1. mean+/- s.e.m, n=3. (*) indicates a pvalue<0.05 (paired t-test).

Biology Open • Supplementary information

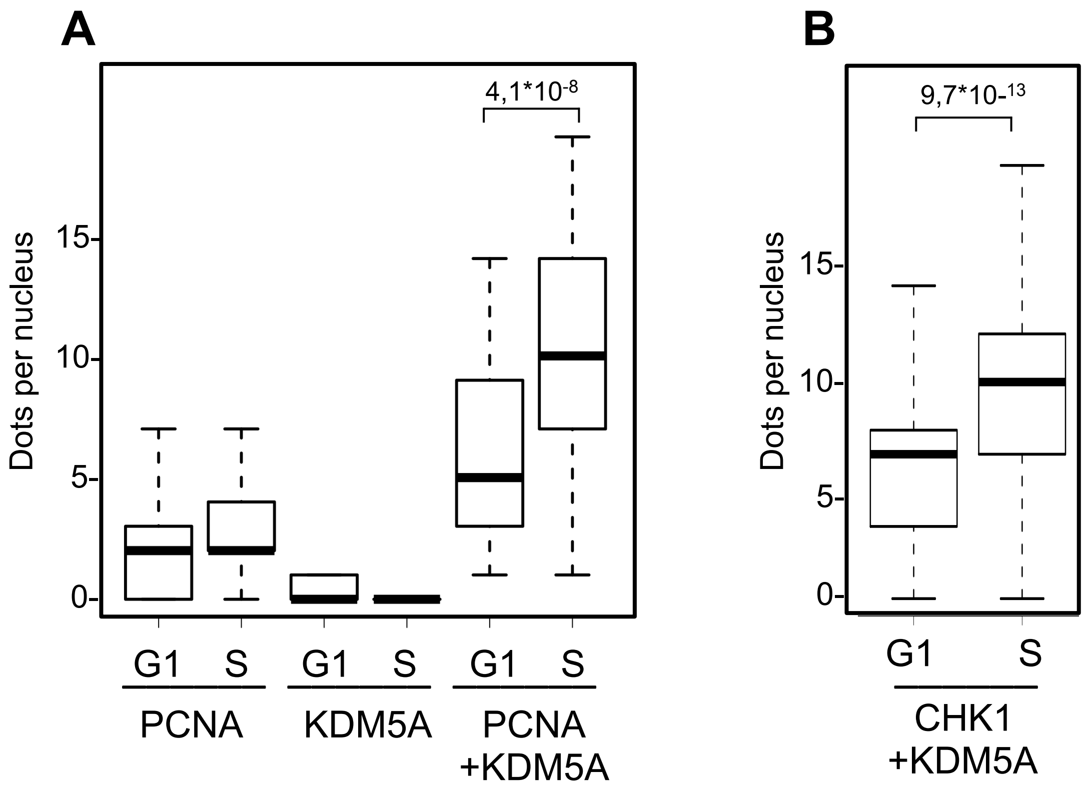

Figure S4. KDM5A interacts with PCNA or CHK1 preferentially in S-phase. A- Proximity ligation assay was performed between KDM5A and PCNA in U2OS cells as in Fig 5D. KDM5A and PCNA antibodies were used either separately or together as indicated. Dots numbers and DAPI staining intensity were quantified for each nucleus using the Colombus software. Cells in G1 and in S were separated according to DAPI staining intensity, and the number of dots per nucleus quantified in G1 and S-phases. Results are presented as a box-plot showing the median, the 25% and 75% quantiles and extrema. PCNA+KDM5A: Number of G1 and S phase nuclei analyzed > 80. pvalue is indicated (Wilcoxon). A representative experiment out of three is shown. **B**- Proximity ligation assay analyzed as in A, except that KDM5A and CHK1 antibodies were used as in Fig 5E. G1 and S-phase cells > 90. the pvalue is indicated (Wilcoxon). A representative experiment out of three is shown.

Biology Open • Supplementary information

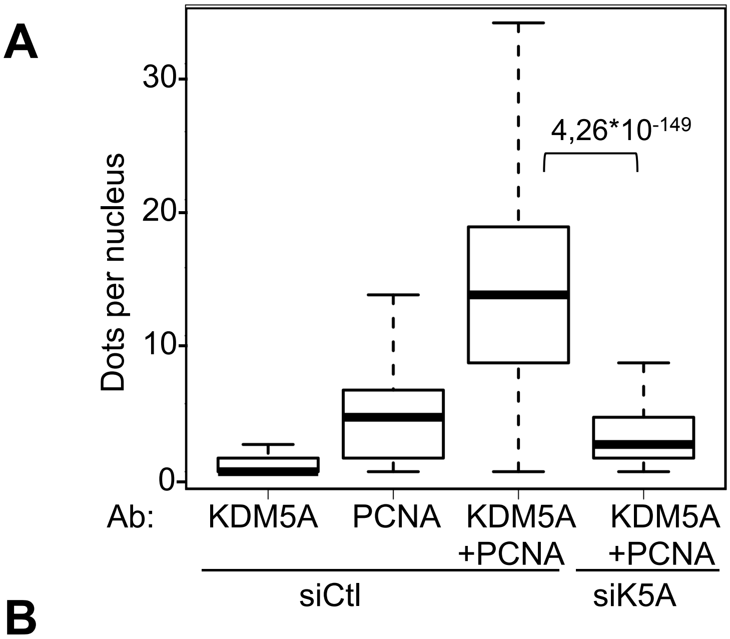

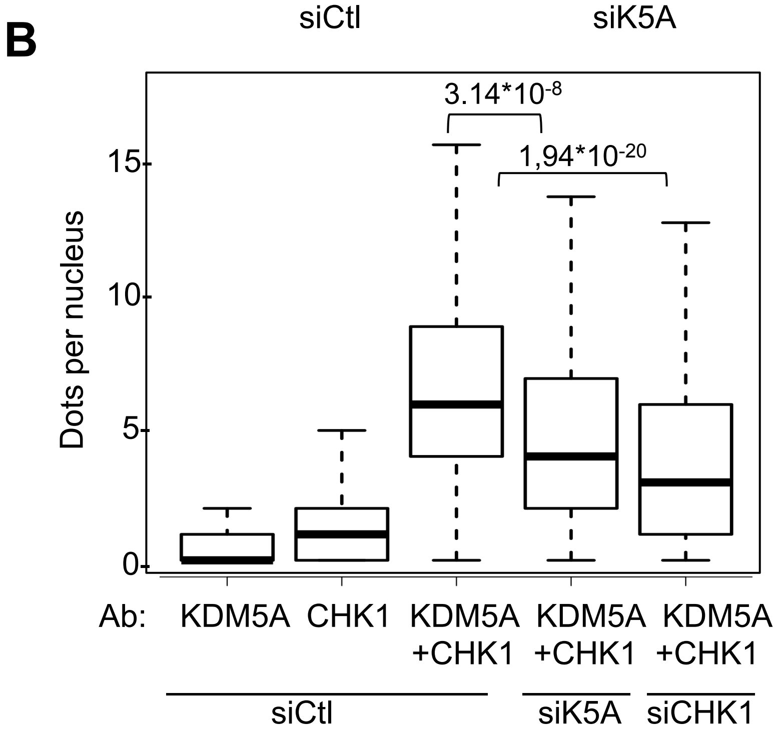

Figure S5. KDM5A specifically interacts with PCNA and CHK1. A- U2OS cells were electroporated with siRNA directed against KDM5A (siK5A) or a non-targeting siRNA as control (siCtl). Antibodies directed against KDM5A or PCNA were used in PLA assay either separately, or mixed (KDM5A+PCNA), as in Figure 5D. PLA signals that appear as dots (not shown) were counted in cell nuclei using the Colombus software. Results are presented as a box-plot showing the median, the 25 % and 75% quantiles and extrema. Number of counted cells is > 500 for each point. pvalue is indicated (Wilcoxon) . A representative experiment out of 3 is shown. **B-** Cells were processed as in A using siCtl, siK5A or siCHK1 siRNA and PLA assay was performed using anti-KDM5A and anti-CHK1 antibodies used either separately or mixed, as in Figure 5E. Number of counted cells is > 500 for each point. pvalues are indicated (Wilcoxon).

Biology Open • Supplementary information

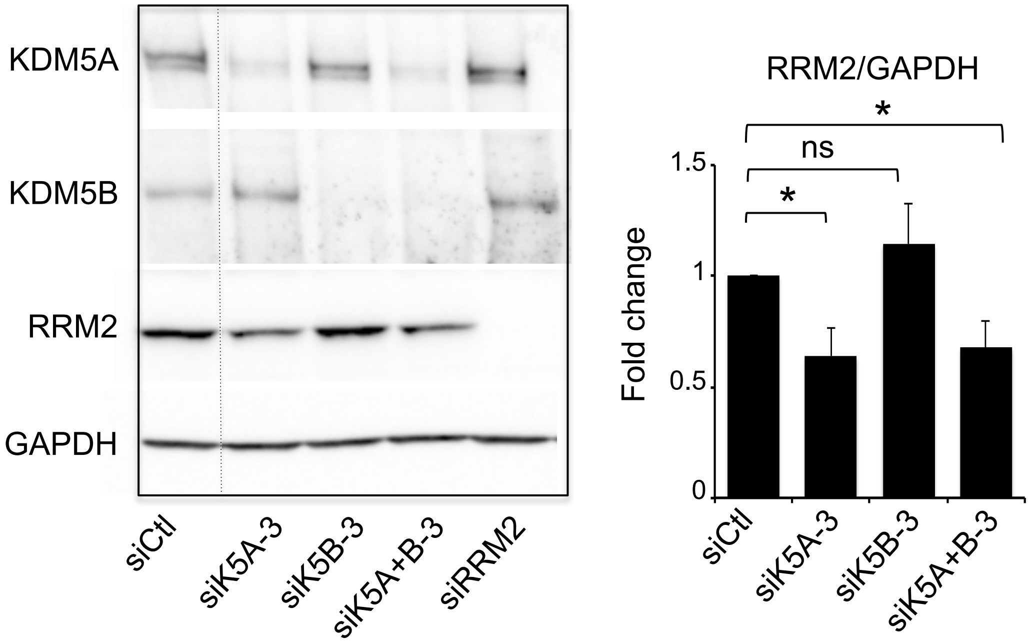

Figure S6. Effect of KDM5A/B depletion on RRM2 protein expression in H50 cells using a second couple of siRNA .

H50 cells cultivated in the presence of 0.5mM HU were treated with siCtl and siKDM5A(siK5A-3) or/and siKDM5B (siK5B-3). 48 hours later, cells were haversted and expression of KDM5A, KDM5B, RRM2 and GAPDH were analyzed by western-blot. **Left panel:** a representative experiment is shown. Right panel: 3 independent experiments were quantified, and the ratio RRM2/GAPDH was calculated and put to 1 for siCtl. mean +/- s.e.m., n=3. * p<0.05 , ns for non significant, using a paired t-test.
